# Variations in Flavonoid Metabolites Along Altitudinal Gradient in a Desert Medicinal Plant *Agriophyllum squarrosum*

**DOI:** 10.3389/fpls.2021.683265

**Published:** 2021-06-30

**Authors:** Shanshan Zhou, Xia Yan, Jian Yang, Chaoju Qian, Xiaoyue Yin, Xingke Fan, Tingzhou Fang, Yuan Gao, Yuxiao Chang, Weimin Liu, Xiao-Fei Ma

**Affiliations:** ^1^Key Laboratory of Stress Physiology and Ecology in Cold and Arid Regions of Gansu Province, Department of Ecology and Agriculture Research, Northwest Institute of Eco-Environment and Resources, Chinese Academy of Sciences, Lanzhou, China; ^2^College of Resources and Environment, University of Chinese Academy of Sciences, Beijing, China; ^3^School of Life Sciences, Nantong University, Nantong, China; ^4^State Key Laboratory Breeding Base of Dao-di Herbs, National Resource Center for Chinese Materia Medica, China Academy of Chinese Medical Sciences, Beijing, China; ^5^Faculty of Materials and Chemical Engineering, Yibin University, Yibin, China; ^6^Agricultural Genomics Institute at Shenzhen, Chinese Academy of Agricultural Sciences, Shenzhen, China; ^7^Zhongnong Haidao (Shenzhen) Biotech Co., Ltd., Shenzhen, China

**Keywords:** *Agriophyllum squarrosum*, flavonoid, targeted metabolic profiling, common garden experiment, local adaptation

## Abstract

*Agriophyllum squarrosum* (L.) Moq., a pioneer plant endemic to the temperate deserts of Asia, could be domesticated into an ideal crop with outstanding ecological and medicinal characteristics. A previous study showed differential flavonoid accumulation between two *in situ* altitudinal ecotypes. To verify whether this accumulation was determined by environmental or genetic factors, we conducted flavonoid-targeted metabolic profiling among 14 populations of *A. squarrosum* collected from regions with different altitudes based on a common garden experiment. Results showed that the most abundant flavonoid in *A. squarrosum* was isorhamnetin (48.40%, 557.45 μg/g), followed by quercetin (13.04%, 150.15 μg/g), tricin (11.17%, 128.70 μg/g), isoquercitrin (7.59%, 87.42 μg/g), isovitexin (7.20%, 82.94 μg/g), and rutin (7.00%, 80.62 μg/g). However, based on a common garden at middle-altitude environment, almost none of the flavonoids was enriched in the high-altitude populations, and even some flavonoids, such as quercetin, tricin, and rutin, were significantly enriched in low-altitude populations. This phenomenon indicated that the accumulation of flavonoids was not a result of local adaptation to high altitude. Furthermore, association analysis with *in situ* environmental variables showed that the contents of quercetin, tricin, and rutin were strongly positively correlated with latitude, longitude, and precipitation gradients and negatively correlated with temperature gradients. Thus, we could conclude that the accumulations of flavonoids in *A. squarrosum* were more likely as a result of local adaption to environmental heterogeneity combined with precipitation and temperature other than high altitude. This study not only provides an example to understand the molecular ecological basis of pharmacognosy, but also supplies methodologies for developing a new industrial crop with ecological and agricultural importance.

## Introduction

As sessile organisms, plants have evolved to produce a variety of chemical metabolites in the process of adaptation to the changing environment, and each metabolite plays a vital role in responding to abiotic stresses. On the other hand, the long-term environmental heterogeneity could exert local selection pressure on plants which increases the fitness of individuals in specific environments (Kawecki and Ebert, 2004). When populations inhabit different environments, divergent selection pressure can result in phenotypic differentiation by metabolite accumulation to confer a local fitness advantage (Kawecki and Ebert, 2004). Normally, when exhibit better fitness in a local population, metabolites, particularly secondary metabolites, should be variated along with environmental gradients or multiple stresses, such as drought, high and low temperature, UV-B, pathogens, and herbivores (Vanwallendael et al., 2019). For example, climatic conditions at a high altitude with higher exposure to UV radiation, lower temperature, and stressed conditions that induce the production of secondary metabolites (Barnes et al., 1987; Turunen and Latola, 2005; Zidorn et al., 2005; Spitaler et al., 2008). Many studies have shown that flavonoid content increased with the increasing altitude (Zidorn et al., 2005; Rieger et al., 2008; Kalim et al., 2010; Macar and Kalefetoğlu Macar, 2018; Rana et al., 2020). However, to date, the contribution of a secondary metabolite involved in the high-altitudinal adaptation based on the common garden experiment has seldom been addressed. In addition, breeding of elite germplasms with local adaptation is an important strategy for mitigating the negative impacts of climate change on ecology and agriculture (Howden et al., 2007; Takeda and Matsuoka, 2008). Thus, it is necessary to elucidate the potential role of secondary metabolites for a plant species to adapt along an environmental gradient.

*Agriophyllum squarrosum* (L.) Moq., also called sand rice, is a pioneer annual psammophyte of the Amaranthaceae family. It is widely distributed in mobile sand and semi-fixed dunes across all the arid and semi-arid regions in Central Asia, the Caucasus, Mongolia, and Siberia, including the Qinghai-Tibet Plateau (Wu et al., 2003). It can survive in extremely high temperatures and drought conditions and can tolerate sand burial (Chen et al., 2014; Li et al., 2015; Zhao et al., 2017). Since the withered plants can reduce wind velocity by more than 90%, they also acted as nutrient providers and reservoirs with a rich source of carbon and nitrogen in infertile soil environments. *A. squarrosum* can effectively prevent wind and fix sand, and plays a crucial role in maintaining and restoring the fragile desert ecosystems (Ma et al., 2008; Chen et al., 2009). Although *A. squarrosum* grows in infertile and sandy soils, its seeds are highly nutritious. Compared with *Chenopodium quinoa*, a world-recognized crop relative with total nutrients (Jacobsen, 2006; Chen et al., 2014), *A. squarrosum* seeds contain lesser quantities of carbohydrates and more protein and crude fiber (Gao et al., 1991; Ren et al., 2005; Yin et al., 2021), and they are also rich in minerals and trace elements, such as calcium, magnesium, iron, zinc, selenium, and potassium (Zhang et al., 2006; Wang et al., 2009). Besides the high nutritious content in its seeds, the young fresh leaves and stems of *A. squarrosum* are suitable for fodder and medical usage (Gao, 2002). *A Supplement to the Compendium of Materia Medica* references that *A. squarrosum* seeds are healthy for the stomach, spleen, and large intestine (Zhao, 1765), and its aboveground parts can be used as antidiabetes, diuresis, analgesic, and antipyretic in Mongolian folk medicine (Gong et al., 2012). Therefore, as a promising crop candidate with pharmacological and agricultural importance, *A. squarrosum* provides an ideal model to understand the molecular ecological basis of pharmacognosy.

Previous studies have indicated significant genetic divergence and phenotypic variations among the populations of *A. squarrosum* (Qian et al., 2016; Yin et al., 2016a,b). Based on common garden trials, the *in situ* environmental heterogeneity was significantly correlated with phenotypic traits and geographical distribution of *A. squarrosum* (Yin et al., 2016a,b). In addition, non-targeted metabonomics analysis showed that *A. squarrosum* is rich in flavonoids (Yin et al., 2018). Flavonoids are ubiquitous plant secondary metabolites that received and continue to receive a great deal of attention in science and medicine, due to the great variety of biological activities and the vast array of biological functions (Winkel-Shirley, 2002). Flavonoids contribute to the adaptation of the plant to environmental stresses, including resistance to diverse abiotic stresses (Liu et al., 2013), such as drought, extreme temperature fluctuations, high soil salinity, and increased exposure to UV radiation (Albert et al., 2018). Moreover, flavonoids play an important role in various plant physiological or developmental functions, as well as in the agronomic and industrial qualities of plant products (Lepiniec et al., 2006; Li et al., 2018). Numerous studies have found that flavonoids have antioxidant, anti-inflammatory, antimicrobial, anti-fungal, antiviral, anti-tumor properties, and also help to scavenge free radicals and stabilize the reactive oxygen species; these keep the body safe from chronic diseases, such as cancer, diabetes, and cardiovascular and heart disease, enhance the immune system, for example, suppress pro-inflammatory immune response, in particular auto-reactive T cells (Havsteen, 2002; Verbeek et al., 2004; Falcone Ferreyra et al., 2012; Mouradov and Spangenberg, 2014; Lei et al., 2019; Sarbu et al., 2019).

Previous studies showed that there was no significant divergence among the altitudinal wild populations in neutral molecular genetic markers (nrITS and chloroplast DNA; Qian et al., 2016, 2021). However, non-target metabolomics analysis supported that the accumulations of a couple of flavonoids, such as hesperetin, quercetin, and apigenin, were significantly enriched in the ecotype with high altitude (unpublished data). Thus, we proposed that the enrichment of flavonoids in the high-altitude populations of *A. squarrosum* was a consequence of physiological response to environmental stresses or of genetic differentiation involved in local adaptation to the high altitude. To test the above hypothesis, we conducted flavonoid-targeted metabolic profiling among 14 populations of *A. squarrosum* based on a common garden experiment, a classic approach for studying local adaptation (Anderson et al., 2010; Fournier-Level et al., 2011; Savolainen et al., 2013). Thus, we aimed to address the following questions: (1) Whether flavonoids are still enriched in the high-altitude populations of *A. squarrosum* at the common garden site. If the results obey the trend, we can conclude that the flavonoid accumulated due to the genetic basis of local adaptation to the high altitude. (2) If not, what other kinds of *in situ* environmental variables influenced the differences in flavonoid metabolites among the populations. Under this circumstance, the differentiation of flavonoids could be determined by the heterogeneity of precipitation and temperature among the populations, which could also be a result of local adaptation for the nature populations. This study could not only shed light on the molecular ecological basis for *A. squarrosum* adapted to the desert heterogeneity, but also provide instructive guidelines for the development and utilization of the wild plant in food, pharmaceutical, and cosmetic industries under global climate change.

## Materials and Methods

### Plant Materials and Common Garden Experiment

**Figure 1:**
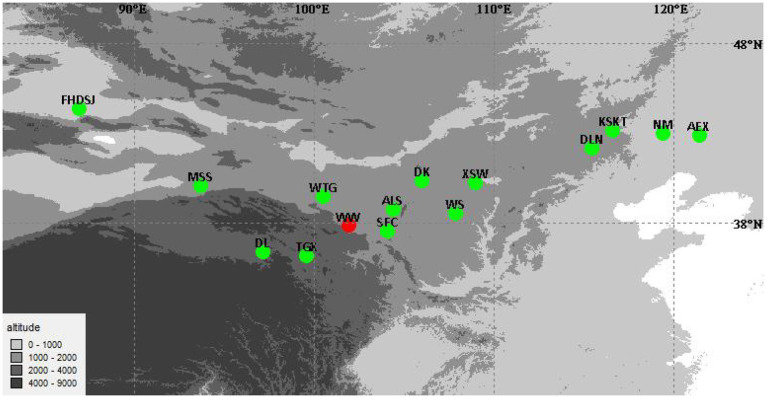
Geographical distribution of 14 *Agriophyllum squarrosum* populations in China. The red dot indicates the planting place of the common garden experiment.

Seeds of 14 wild *A. squarrosum* populations, representing the different altitude regions of *A. squarrosum* in China, were sampled in 2016 (Figure 1; Table 1). According to the altitude, the 14 *A. squarrosum* populations were divided into three groups: low-altitude (~0–1,000 m), middle-altitude (~1,000–2,000 m), and high-altitude (~2,000–4,000 m) groups. To identify the accumulation of flavonoid metabolites in these *A. squarrosum* populations, a common garden experiment was conducted in natural environmental conditions in Wuwei City, Gansu Province (WW, 37°54′10.98″N, 102°54′4.2″E, 1530 m) at the southern edge of the Tengger Desert (Figure 1). Seeds of *A. squarrosum* were sown on April 20, 2018, and seedlings initially raised in a nursery bed were transplanted into the common garden after 10 days. Twenty randomly selected seedlings of each population were planted at 2-m intervals. Fresh tissues of *A. squarrosum*, including leaves, stems, and spikes, were randomly collected from six individuals of each population in early August 2018 at the flowering phase. All the materials were frozen immediately in liquid nitrogen and then stored at −80°C for further use.

**Table 1:** Details of the locality information of the sampled populations.

Code	Population code	Altitude-categories	Location (All in China)	Latitude (N°)	Longitude (E°)	Altitude (m)	Annual precipitation (mm)	Annual mean temperature(°C)
1	DL	High	Dulan, Qaidam	36°25'25.77''	98°7'25.35''	3,130	263	3.40
2	TGX		Tiegaixiang, Qaidam	36°10'02.09''	100°34'13.85''	2,911	321	3.08
3	SFC	Middle	Shapotoushuifenchang, Tengger	37°32'33.58''	105°02'10.14''	1,297	191	9.45
4	WTG		Wutonggou, Gurbantunggut	39°30'12.96''	101°28'54.84''	1,489	103	7.33
5	ALS		Alxa League, Tengger	38°45'36.12''	105°22'14.64''	1,301	168	8.79
6	WS		Wushenqi, Mu Us	38°33'23.36''	108°51'6.05''	1,286	352	7.98
7	DLN		Duolunnan, Hunshadake	42°9'20.63''	116°27'55.14''	1,269	383	2.10
8	XSW		Xiangshawan, Kubuqi	40°14'29.16''	109°57'45.12''	1,181	343	6.31
9	MSS		Mingshashan, Dunhuang	40°5'41.73''	94°39'11.73''	1,155	37	9.74
10	KSKT		Kesheketeng, Hunshadake	43°10'28.8''	117°34'48''	1,051	383	3.13
11	DK		Dengkou, Ulanbuh	40°22'42.30''	106°59'37.51''	1,050	137	8.31
12	FHDSJ	Low	Fuhedashajiao, Gurbantunggut	44°21'49.69''	87°53'9.82''	461	172	8.36
13	NM		Naiman, Horqin	42°52'29.68''	120°38'46.77''	370	386	6.83
14	AEX		Aerxiang, Horqin	42°52'4.80''	122°25'40.14''	251	485	6.38
	WW	Middle	Wuwei, Tengger	37°54'10.98''	102°54'4.2''	1,530	177	8.23

### Flavonoid-Targeted Metabolic Profiling

The samples of *A. squarrosum* were dried at 60°C in a drying oven after washing. For each sample, 50 mg homogenized sample powder was suspended in 1.5 ml 70% v/v ethanol overnight. Then, the extract solution was extracted by ultrasonication for 30 min at a 40 KHz SCIENT ultrasonic processor (Ningbo, China). The extract was centrifuged for 10 min at 12,000 × *g* and the supernatant was subsequently filtered through a 0.22-μm syringe filter for UPLC-QQQ-MS/MS analysis. Chromatographic analysis was performed using the Acquity UPLC^T^ I-Class system (Waters, Milford, MA, United States). The column used was Acquity UPLC BEH C18 column (2.1 mm × 100 mm, 1.8 μm), and the column temperature was maintained at 40°C. The binary gradient consisted of solvent system A (formic acid/water, 0.1:99.9, v/v) and solvent system B (formic acid/acetonitrile, 0.1:99.9, v/v). The chromatographic conditions were as follows: 0 min, 5% B; 1.0 min, 25% B; 3.5 min, 40% B; and 4.5 min, 60% B. The injection volume was 1.0 μl, and the flow rate was 0.60 ml/min. Tandem mass spectrometry (MS/MS) was performed using QTRAP 6500 system (AB SCIEX, Los Angeles, CA, United States) equipped with an electrostatic ionization (ESI) source (AB SCIEX). The MS spectra were acquired in negative ion mode, which was carried out by optimization of the product ion obtained from the fragment of the isolated precursor ion for each analyte. The ion spray potential was −4,500 V, and the source temperature was set at 550°C. After the product ions were chosen, the multiple reaction monitoring conditions for each standard were further optimized to achieve maximum sensitivity. The retention times (*t*_R_), quantitative ion pairs, declustering potential (DP), collision energy (CE), and cell exit potential (CXP) are listed in Table 2.

**Table 2:** Mass spectra properties of flavonoids evaluated in *A. squarrosum*.

Compounds	Retention (min)	Quantitative ion pairs (*m/z*)	DP (V)	CE (V)	CXP (V)
Rutin	1.41	609.1/300.0	−245	−48	−38
Orientin	1.36	447.1/327.0	−173	−30	−27
Isovitexin	1.46	431.1/311.2	−11	−37	−27
Tricin	2.49	329.0/151.0	−148	−28	−12
Isoquercitrin	1.48	463.1/300.0	−191	−36	−22
Luteoloside	1.51	447.1/285.0	−146	−34	−25
Isorhamnetin 3-*O*-glucoside	1.56	477.0/313.9	−14	−37	−21
Avicularin	1.57	433.0/301.0	−15	−27	−32
Astragaline	1.61	447.1/254.8	−170	−46	−23
Eriodictyol	2.20	287.0/151.0	−71	−21	−12
Luteolin	2.24	285.0/133.0	−144	−40	−15.2
Quercetin	2.25	301.0/159.9	−82	−53	−19
Naringenin	2.72	271.0/150.9	−85	−25	−17
Kaempferol	2.81	285.0/210.7	−30	−46	−23
Chrysoeriol	2.88	299.1/284.0	−160	−27	−33
Hesperitin	2.96	300.8/164.0	−89	−33	−17
Isorhamnetin	3.00	315.0/299.8	−25	−29	−22
Chrysin	4.21	253.0/143.0	−147	−38	−17
Pinocembrin	4.32	255.0/171.0	−94	−36	−11
Biochanin A	4.42	283.0/268.0	−89	−30	−18

DP, declustering potential; CE, collision energy; CEP, cell exit potential.

### Environmental Variables

To test the correlations of different flavonoid metabolites with environmental variables, we extracted 22 environmental variables of the original locations of the 14 *A. squarrosum* populations from the world climate database^1^ (Supplementary Table S1), including altitude, annual mean temperature (AMT), mean monthly temperature range (MMTR), isothermality, temperature seasonality (TS), maximum temperature of the warmest month (MATWM), minimum temperature of the coldest month (MITCM), temperature annual range (TAR), mean temperature of the wettest quarter (MTWEQ), mean temperature of the driest quarter (MTDQ), mean temperature of the warmest quarter (MTWAQ), mean temperature of the coldest quarter (MTCQ), annual precipitation (AP), precipitation of the wettest month (PWM), precipitation of the driest month (PDM), precipitation seasonality (PS), precipitation of the wettest quarter (PWEQ), precipitation of the driest quarter (PDQ), precipitation of the warmest quarter (PWAQ), precipitation of the coldest quarter (PCQ), longitude, and latitude.

### Statistical Analyses

To determine the content differences in flavonoids among the examined groups, one-way ANOVA, Tukey honest significant difference (HSD) test, and pairwise Pearson’s correlations were performed using IBM SPSS Statistics (version 25; IBM Corporation, Armonk, NY, United States) and OriginPro2020 (OriginLab Corporation, Northampton, MA, United States). To discern the similarities and differences in the profiles of the flavonoids among these *A. squarrosum* populations, principal component analysis (PCA) was performed using SIMCA software (version 13.0; Umetrics AB, Umeå, Sweden). To maximize the separation between the different groups, the supervised models were subsequently formulated by orthogonal partial least-squares discrimination analysis (OPLS-DA). To show the variation in the flavonoid metabolites in different *A. squarrosum* populations, a cluster heatmap was implemented by the heatmap package in R (version 1.1.463; RStudio, Boston, MA, United States). To investigate the relationship between environmental variables and flavonoid metabolites in *A. squarrosum*, univariate linear regression analysis was performed using OriginPro2020.

## Results

### Composition and Content of Flavonoid Metabolites in *A. squarrosum*

**Figure 2:**
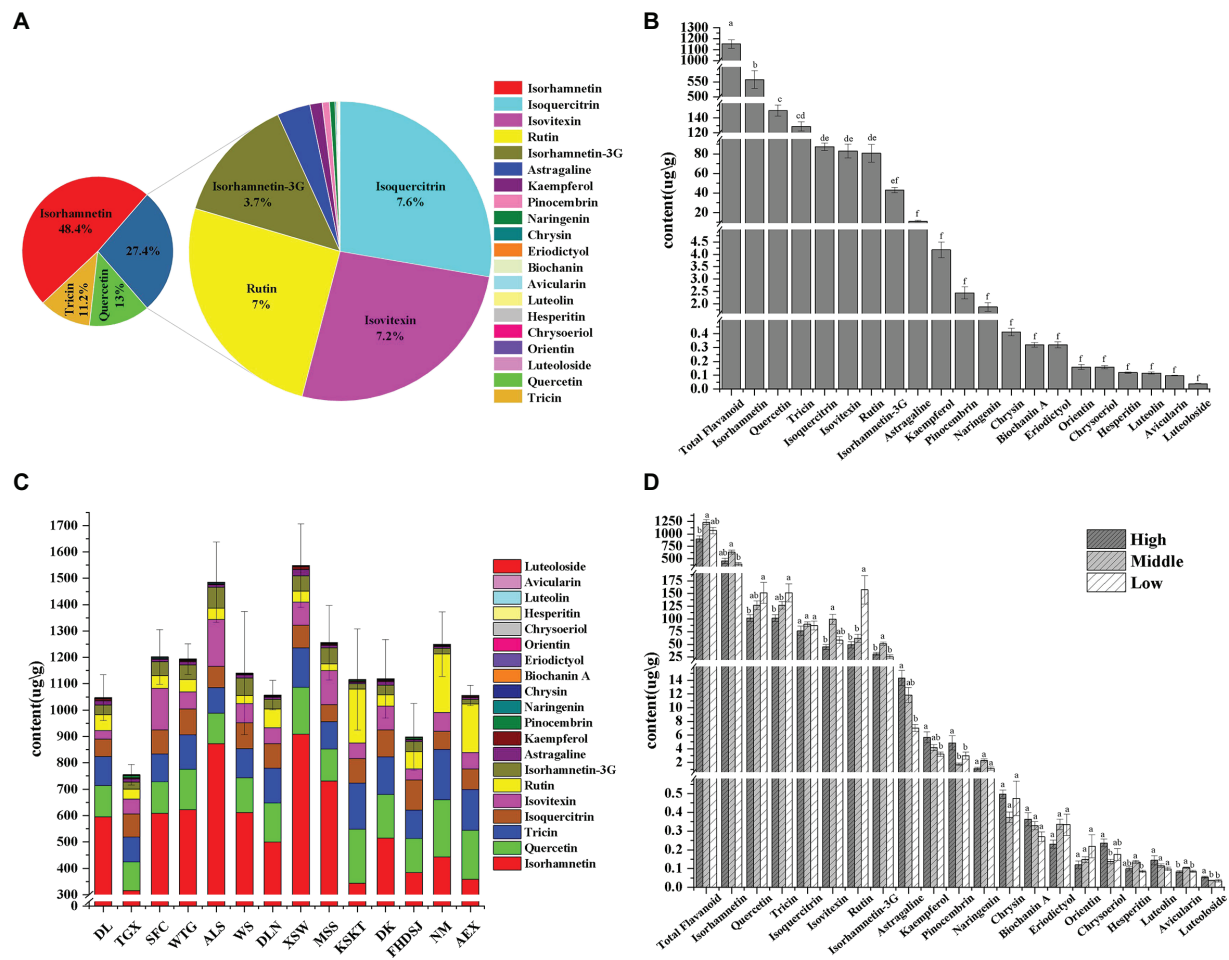
Flavonoid content in *A. squarrosum*. The composition **(A)** and content **(B)** of flavonoids in *A. squarrosum*. Values are Mean ± SE (*n* = 84). **(C)** The composition and content of flavonoids in 14 *A. squarrosum* populations. Values are Mean ± SE (*n* = 6). **(D)** The composition and content of flavonoids in different altitude populations. Values are Mean ± SE (*n* = 12, 54, 18). Values marked with the different or same letters indicate statistically significant and not significant differences, respectively, using Tukey honest significant difference (HSD) test at the 5% significance level.

As shown in Figure 2 and Supplementary Table S2, 20 flavonoid metabolites were identified from 14 *A. squarrosum* populations. Figures 2A,B show the averages from 14 *A. squarrosum* populations. The flavonoid metabolites content in *A. squarrosum* varied from 0.04 μg/g (luteoloside, 0.003%) to 557.45 μg/g (isorhamnetin, 48.40%). Quercetin (13.04%, 150.15 μg/g), tricin (11.17%, 128.70 μg/g), isoquercitrin (7.59%, 87.42 μg/g), isovitexin (7.20%, 82.94 μg/g), rutin (7.00%, 80.62 μg/g), and isorhamnetin-3-glucoside (3.73%, 43.01 μg/g) were highly accumulated in *A. squarrosum* (Figures 2A,B; Supplementary Table S2b).

Among the different populations, the total flavonoid content ranged from 754.74 μg/g [population in Tiegaixiang, Qaidam (TGX)] to 1548.62 μg/g [population in Xiangshawan, Kubuqi (XSW)], averaging 1151.70 μg/g (Figure 2C; Supplementary Table S2c). The level of quercetin, tricin, and rutin stood out with the higher content in low-altitude group than in the high- and middle-altitude groups (Figure 2D; Supplementary Table S2d).

### Differences in Flavonoid Metabolites Among *A. squarrosum* Populations

**Figure 3:**
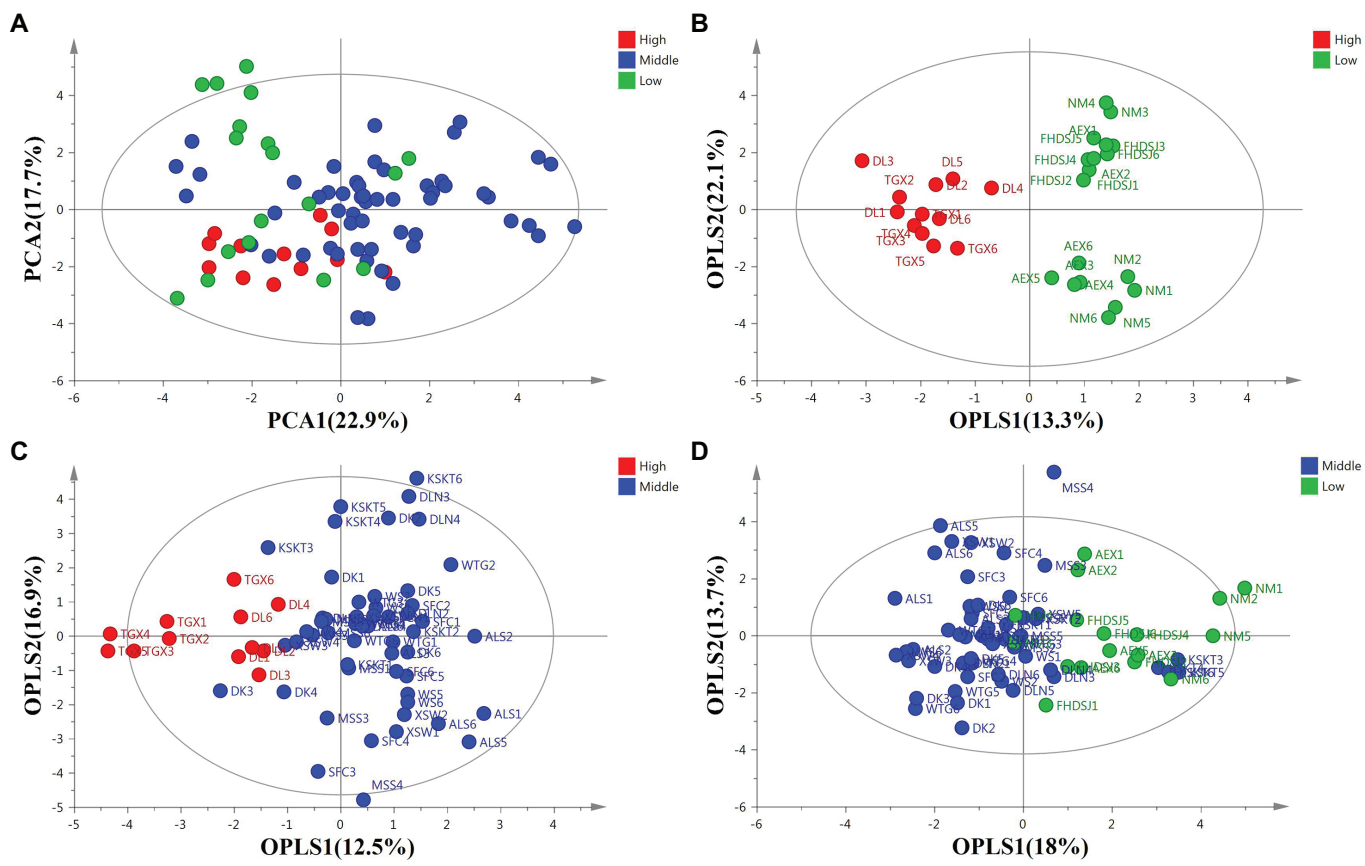
Score plots of flavonoid metabolites in *A. squarrosum*. **(A)** Principal component analysis score plot of total samples (*R*^2^ = 0.559, *Q*^2^ = 0.213). **(B)** Orthogonal partial least-squares discrimination analysis (OPLS-DA) score plot of high-altitude vs. low-altitude sample (R^2^X = 0.714, R^2^Y = 0.917, *Q*^2^ = 0.777). **(C)** OPLS-DA score plot of high-altitude vs. middle-altitude sample (R^2^X = 0.422, R^2^Y = 0.586, *Q*^2^ = 0.397). **(D)** OPLS-DA score plot of middle-altitude vs. low-altitude sample (R^2^X = 0.316, R^2^Y = 0.404, *Q*^2^ = 0.227). Green, blue, and red circles indicate sample sites of low-altitude (~0–1,000 m), middle-altitude (~1,000–2,000 m), and high-altitude (~2,000–4,000 m), respectively.

After a comprehensive view of the similarities and differences in the profiles of the flavonoids among *A. squarrosum* populations, PCA and OPLS-DA were performed. The unsupervised PCA model was also performed to observe the trends of flavonoid metabolites in *A. squarrosum* (Figure 3A). The principal components 1 (PC1, 22.9%) and 2 (PC2, 17.7%) explain 45.2% of the metabolic variance among samples. As shown in Figure 3A, PCA did not show a clear separation among the three altitude groups. To identify the variables contributing to the separation between the two sample groups, a supervised OPLS-DA was performed. OPLS-DA between the high-altitude and low-altitude groups gave a strong model with *R*^2^ = 0.714 and *Q*^2^ = 0.777, and the plot showed complete separation of the two groups (Figure 3B). The total variation in the high-altitude group vs. the low-altitude group was 35.6%, of which 13.3% was related to the separation between the high-altitude and low-altitude groups, while 22.1% was within group variation. OPLS-DA between the high-altitude and middle-altitude groups gave a good model with *R*^2^ = 0.422 and *Q*^2^ = 0.586, and the plot showed definite groupings with a small overlap (Figure 3C). Class separation was not as clear as in the model middle-altitude vs. low-altitude sample (Figure 3D). These results indicated that the accumulation of 20 flavonoids was found to be significantly different between the high-altitude group and the other two altitude groups, while not significantly different between the middle-altitude and low-altitude groups.

**Figure 4:**
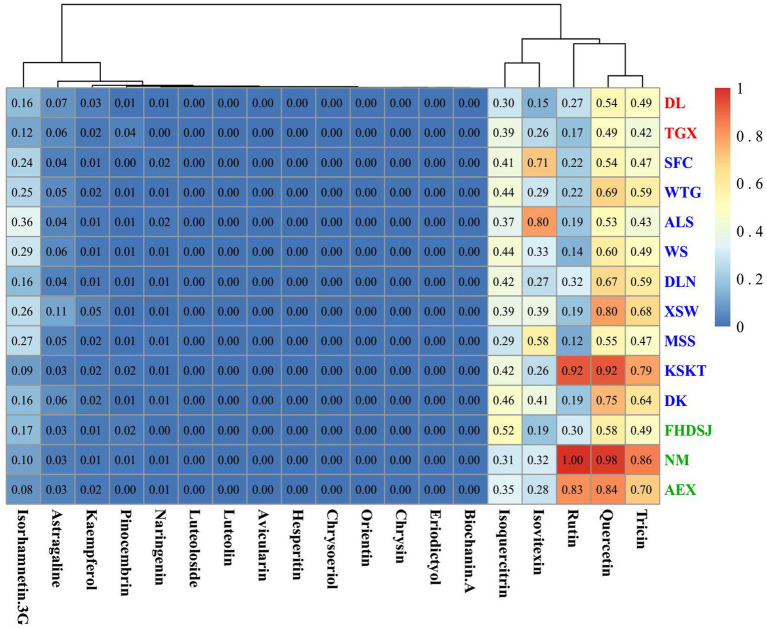
Cluster heatmap of flavonoid metabolites in *A. squarrosum*. Metabolites and populations two-way clustering heatmap. Color depth represents average intensity of metabolite contents in 14 populations of *A. squarrosum*. Red represents highest content and blue represents lowest content. Altitude categories are encoded using three colors: green, blue, and red indicates sample sites of low-altitude (~0–1,000 m), middle-altitude (~1,000–2,000 m), and high-altitude (~2,000–4,000 m), respectively.

The level of isorhamnetin was more than 2-fold higher than that of the other 19 flavonoid metabolites, and this prevented the cluster heatmap from showing the differences in flavonoid metabolites in the different populations. So, we produced the cluster heatmap of 19 flavonoid metabolites except for isorhamnetin among 14 *A. squarrosum* populations. Figure 4 shows that the content of flavonoid metabolites varied among different populations. As shown in Figure 4, the 19 flavonoid metabolites were clustered into three distinct metabolite groups. The first group contained high levels of three flavonoid metabolites, including quercetin, tricin, and rutin. The second group contained medium levels of two flavonoid metabolites, including isoquercitrin and isovitexin. The last group contained low levels of 14 flavonoids, such as isorhamnetin-3-glucoside, astragaline, and so on.

As shown in Figure 4, populations in Dulan, Qaidam, and TGX of the high-altitude group had lower values in most of the measured flavonoids. The middle-altitude group was characterized by medium levels and the low-altitude group had high values in most of the measured flavonoids. These results indicated that most flavonoid metabolites, especially quercetin, tricin, and rutin, were more in the low-altitude populations than in the high- and middle-altitude groups (Figures 2D, 4).

### Relationship Between Environmental Variables and Flavonoid Metabolites in *A. squarrosum*

**Figure 5:**
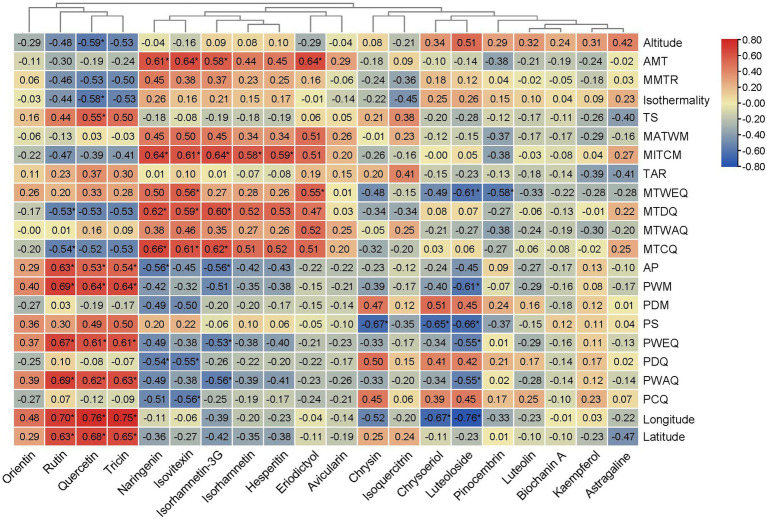
Cluster heatmap based on correlation between flavonoid metabolites and the environmental variables. Red represents positive correlation, blue represents negative correlation. *Indicates statistical significance (*p* < 0.05).

Univariate linear regression was implemented to identify the major *in situ* environmental variables determining the flavonoid metabolites in the *A. squarrosum* (Figure 5; Supplementary Figures S1–S22). We found that quercetin, tricin, and rutin were strongly positively correlated with latitude, longitude, and precipitation gradients, such as AP, PWAQ, PWEQ, and PWM. While rutin was significantly negatively correlated with temperature gradients, such as MTDQ and mean MTCQ, quercetin was significantly negatively correlated with isothermality. Naringenin, isovitexin, and isorhamnetin-3-glucoside were mainly positively correlated with temperature gradients, such as AMT, MITCM, MTDQ, and MTCQ. Isorhamnetin and hesperitin had significantly positive correlationship with MITCM. Eriodictyol had a significantly positive correlationship with AMT and MTWEQ. These results indicated that naringenin, isovitexin, isorhamnetin-3-glucoside, hesperitin, and eriodictyol were mainly positively correlated with temperature, while rutin and quercetin were significantly negatively correlated with temperature.

## Discussion

Flavonoids play an important role in the agronomic and industrial qualities of plant products with tremendous medicinal value (Lepiniec et al., 2006; Li et al., 2018; Sarbu et al., 2019). Our results showed that isorhamnetin was the most abundant flavonoid among 20 identified flavonoid metabolites in *A. squarrosum*, accounted for more than 48.40%, averaging 0.56 mg/g in our study (Figures 2A,B), which is equivalent to the isorhamnetin content in another medicinal plant kale (Schmidt et al., 2010). Isorhamnetin has a wide range of pharmacological effects on cardiovascular, hyperuricemia, and pulmonary fibrosis diseases and a variety of tumors, and possesses the potential of preventing neurodegenerative diseases such as Alzheimer’s (Gong et al., 2020). As an annual medicinal psammophyte with large aboveground biomass, *A. squarrosum* provides ecological benefits of windbreak and sand fixation, making good use of barren sandy land. So, *A. squarrosum* possesses great potential for promotion and application, and its high isorhamnetin content makes it attractive to the pharmaceutical and food industries.

As sessile organisms, plants are often exposed to various environmental stress factors. Among them, abiotic stresses (such as cold, heat, drought, salinity, and UV radiation) are major constraints that affect plant development and growth and pose serious threats to plants’ life. Hence, plants must regulate their growth and development in response to abiotic stresses through primary and secondary metabolism (Cetinkaya et al., 2016). Some secondary metabolite accumulation was positively influenced by abiotic stress conditions, as a consequence of plant adaptive strategies concerned with the establishment of some changes allowing the plant to sustain its life under abiotic stress conditions. Among them, flavonoids could respond to the different stressors, which seems a universal mechanism that flavonoid accumulation is enhanced under abiotic stress (Vanderauwera et al., 2005; Walia et al., 2005; Sun et al., 2020). On the other hand, long-term selection can lead to the development of morphological and physiological adaptations to the local environment, generating genetic differentiation in important traits, including specific metabolites (Kawecki and Ebert, 2004; Savolainen et al., 2007). Individuals within a species may diversify by orders of magnitude in chemical constituents, sizes, and reproductive and growth rates (Callaway et al., 2003). The processes involved in the metabolism and accumulation of chemical constituents in plants are under both genetic and environmental control. Therefore, it is necessary to conduct a common garden experiment across the populations with heterogeneity, which could minimize the environmental effect on the accumulation of the metabolites.

*Agriophyllum squarrosum* populations distributed across a wide range of environmental conditions are subjected to differential selective pressures. Our recent study showed that flavonoid metabolites were significantly enriched in the *in situ* high-altitude ecotype than in the *in situ* middle-altitude ecotypes of *A. squarrosum* (unpublished data). However, in this common garden experiment, we did not find that flavonoid metabolites were significantly enriched in the high-altitude populations, and even some flavonoids, such as quercetin, tricin, and rutin, were significantly enriched in the low-altitude populations (Figures 2D, 4). Considering that the common garden experiment was performed in the middle-altitude environment where the environmental stress for the high-altitude population was decreased, but environmental stress for the low-altitude populations was increased, accordingly, flavonoids were significantly enriched in the low-altitude populations than in the high- and middle-altitude populations. Thus, we proposed that the accumulation of flavonoids was not related to local adaptation to high altitude.

Based on stress treatment, some studies showed that low temperature would increase the accumulation of quercetin (Albert et al., 2009; Jaakola and Hohtola, 2010). However, another study found that high temperature can increase rutin concentration (Lumingkewas et al., 2015). Some field sampling studies have found that the concentration of quercetin derivates correlated positively with the *in situ* latitude (Stark et al., 2008; Sun et al., 2020). Theoretically, the environment of parents may influence characters, such as phenotype and physiological activity, in the offspring (Galloway, 2001). However, without common garden experiments across populations, it is hard to eliminate the environmental imprint. In this study, through investigating the correlations of 20 flavonoid metabolites with environmental variables from the original locations of 14 *A. squarrosum* populations, we detected that quercetin, tricin, and rutin were strongly positively correlated with latitude, longitude, and precipitation gradients, such as AP, PWAQ, PWEQ, and PWM. While rutin was significantly negatively correlated with temperature gradients, such as MTDQ and MTCQ; quercetin was significantly negatively correlated with isothermality (Figure 5). This phenomenon suggested that flavonoids accumulated among the populations of *A. squarrosum* as a result of natural selection of environmental heterogeneity. Of course, more treatment experiments should be conducted to verify this point. These findings could guide us to screen the germplasm and planting field with proper precipitation and temperatures to guarantee maximized yield of flavonoid in *A. squarrosum*.

## Conclusion

This study showed a high variation of the 20 measured flavonoid metabolites among 14 *A. squarrosum* populations from different altitudinal regions based on a common garden experiment. Among them, the contents of quercetin, tricin, and rutin were significantly enriched in low-altitude populations than in middle- and high-altitude, which were positively correlated to precipitation of the *in situ* habitat. This study could shed light on guidelines for future breeding and field management of *A. squarrosum*.

## Data Availability Statement

The original contributions presented in the study are included in the article/Supplementary Material, further inquiries can be directed to the corresponding authors.

## Author Contributions

SZ: writing–original draft, software, and visualization. JY, XYi, XF, TF, and YG: investigation. CQ, YC, and WL: writing–review and editing. X-FM and XYa: conceptualization, methodology, supervision, and writing–review and editing. All authors contributed to the article and approved the submitted version.

### Conflict of Interest

WL was employed by the company Zhongnong Haidao (Shenzhen) Biotech Co., Ltd.The remaining authors declare that the research was conducted in the absence of any commercial or financial relationships that could be construed as a potential conflict of interest.

## Footnotes

^1^www.worldclim.org
