# Supplementary material for: Variations in Flavonoid Metabolites Along Altitudinal Gradient in a Desert Medicinal Plant Agriophyllum squarrosum
Source: Front Plant Sci. 2021 Jun 30;12:683265. doi: 10.3389/fpls.2021.683265 (PMC8329721; doi:10.3389/fpls.2021.683265)
Supplement: Supplementary file 2 [file Image_1.pdf]

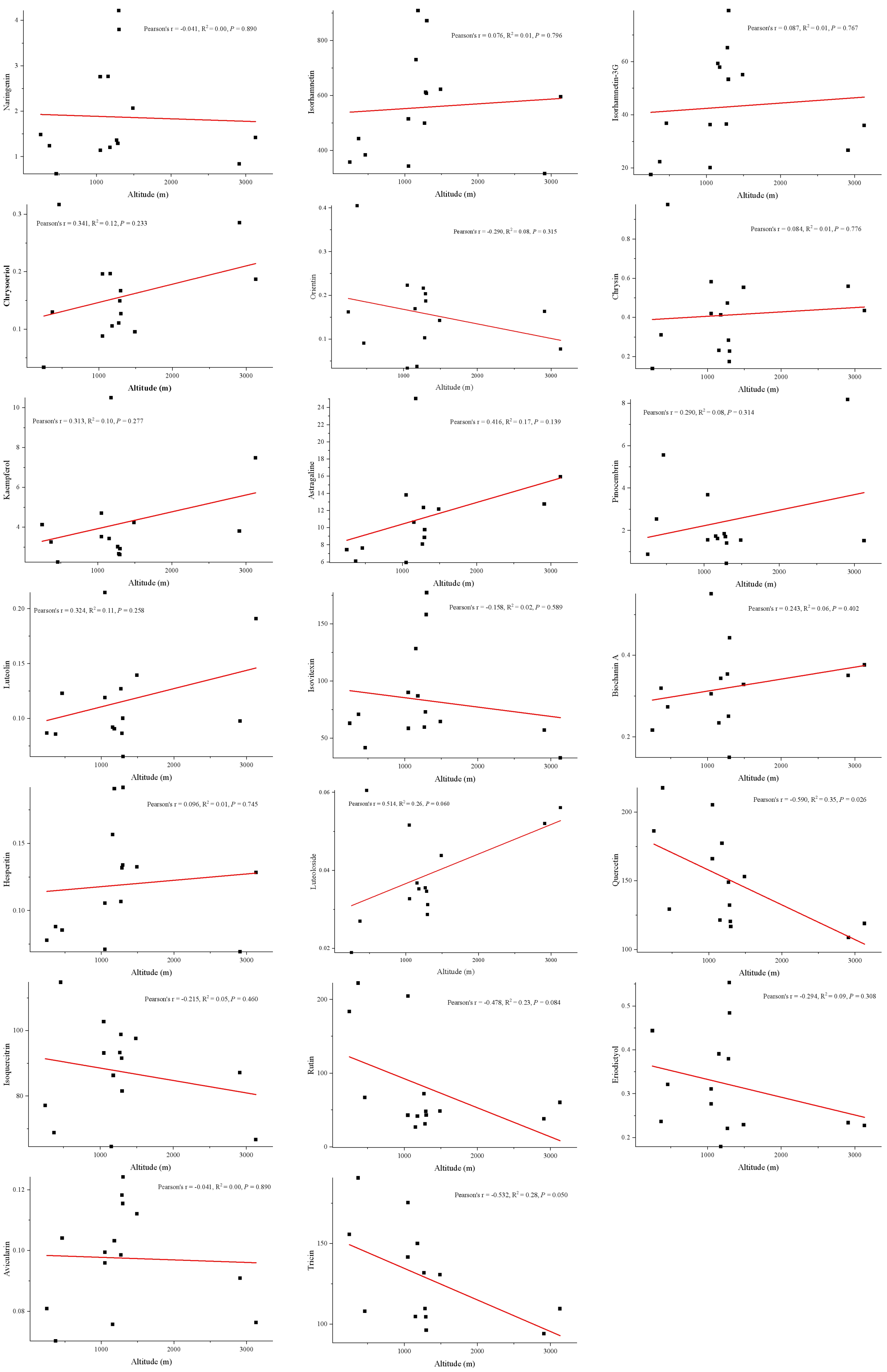

Figure S1 Relationship between altitude and flavonoid metabolites. The linear regressions were considered significant when $P < 0.05$.

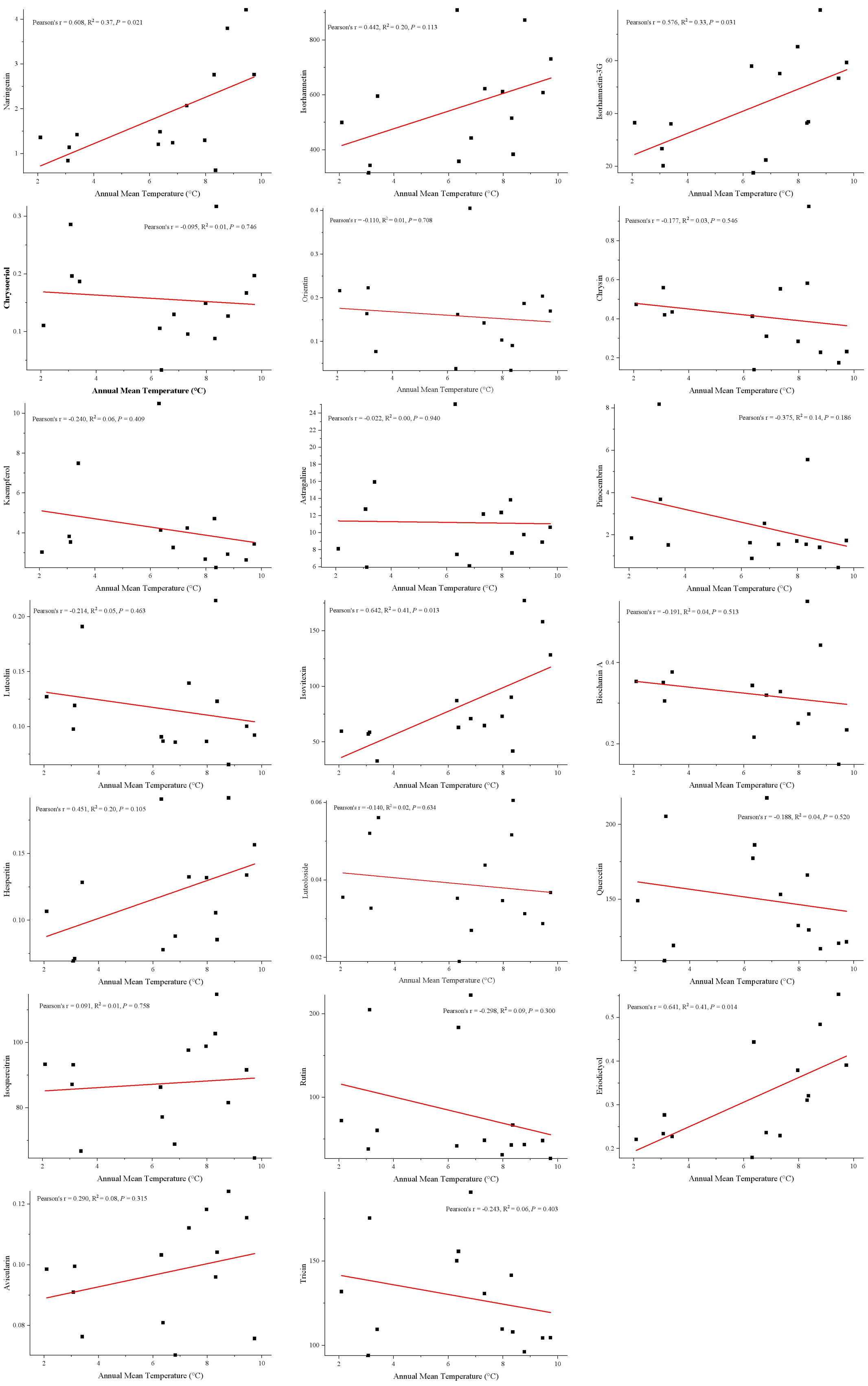

Figure S2 Relationship between annual mean temperature and flavonoid metabolites. The linear regressions were considered significant when $P < 0.05$.

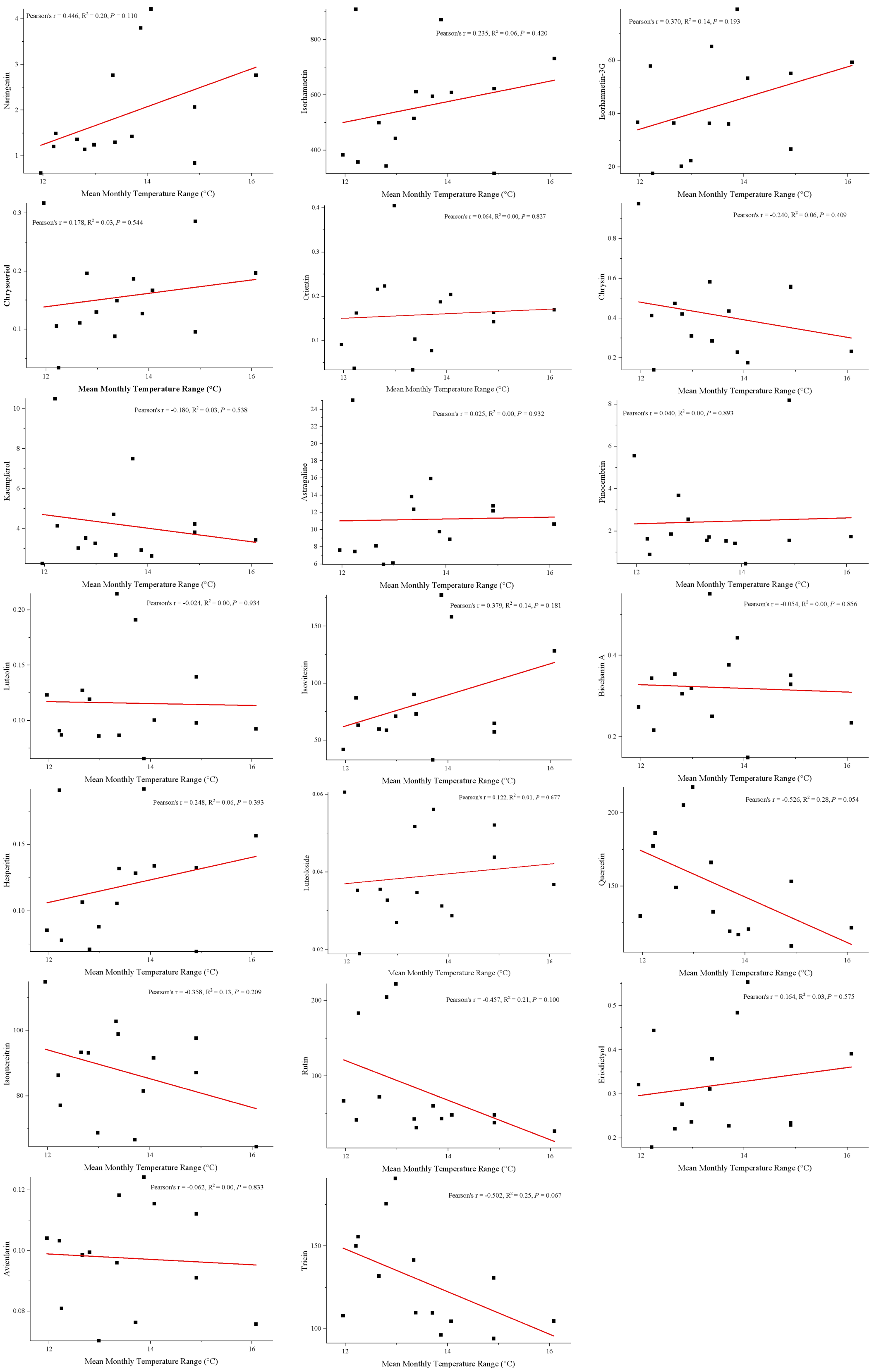

Figure S3 Relationship between mean monthly temperature range temperature and flavonoid metabolites. The linear regressions were considered significant when $P < 0.05$.

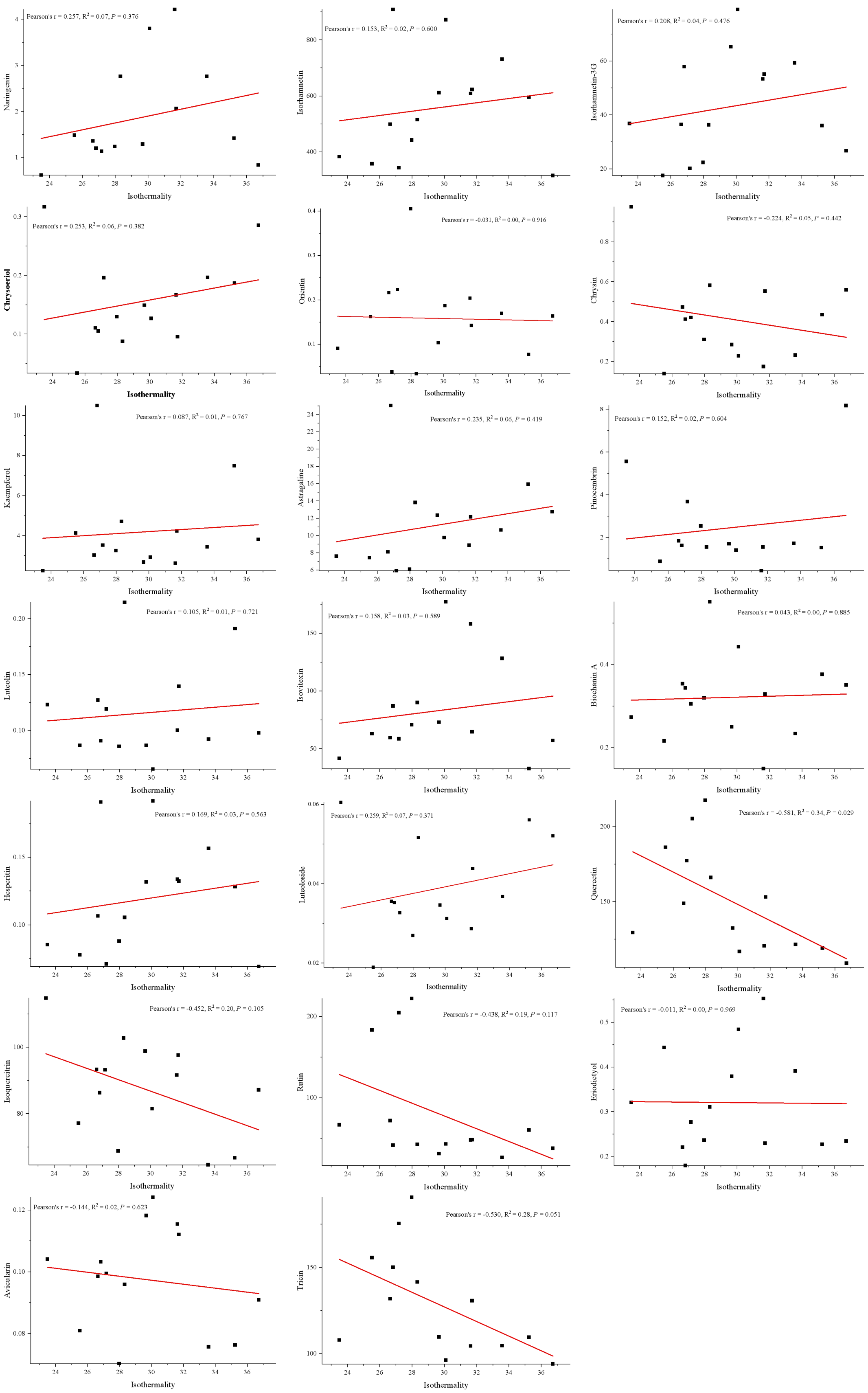

Figure S4 Relationship between Isothermality and flavonoid metabolites. The linear regressions were considered significant when $P < 0.05$.

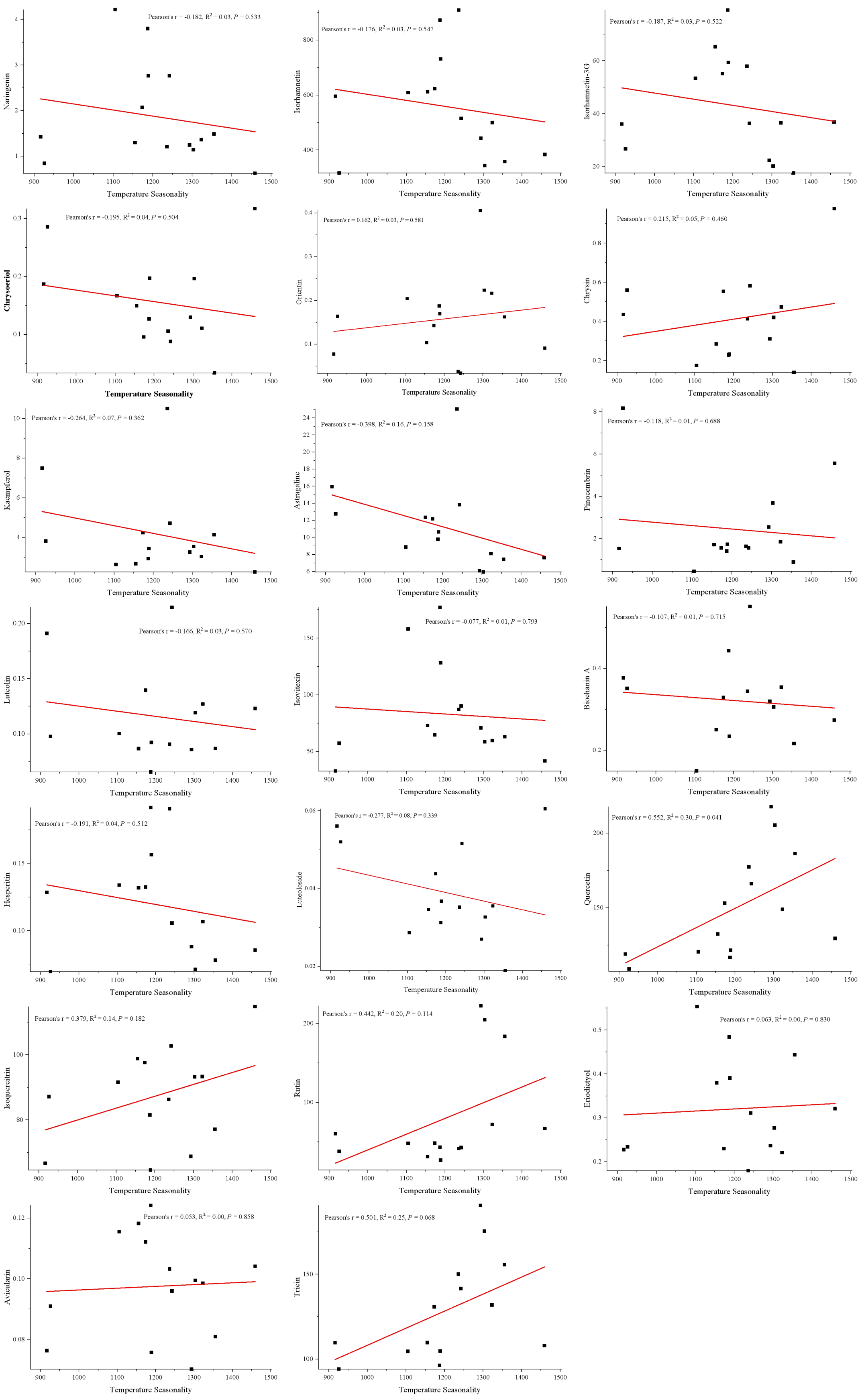

Figure S5 Relationship between temperature seasonality and flavonoid metabolites. The linear regressions were considered significant when $P < 0.05$.

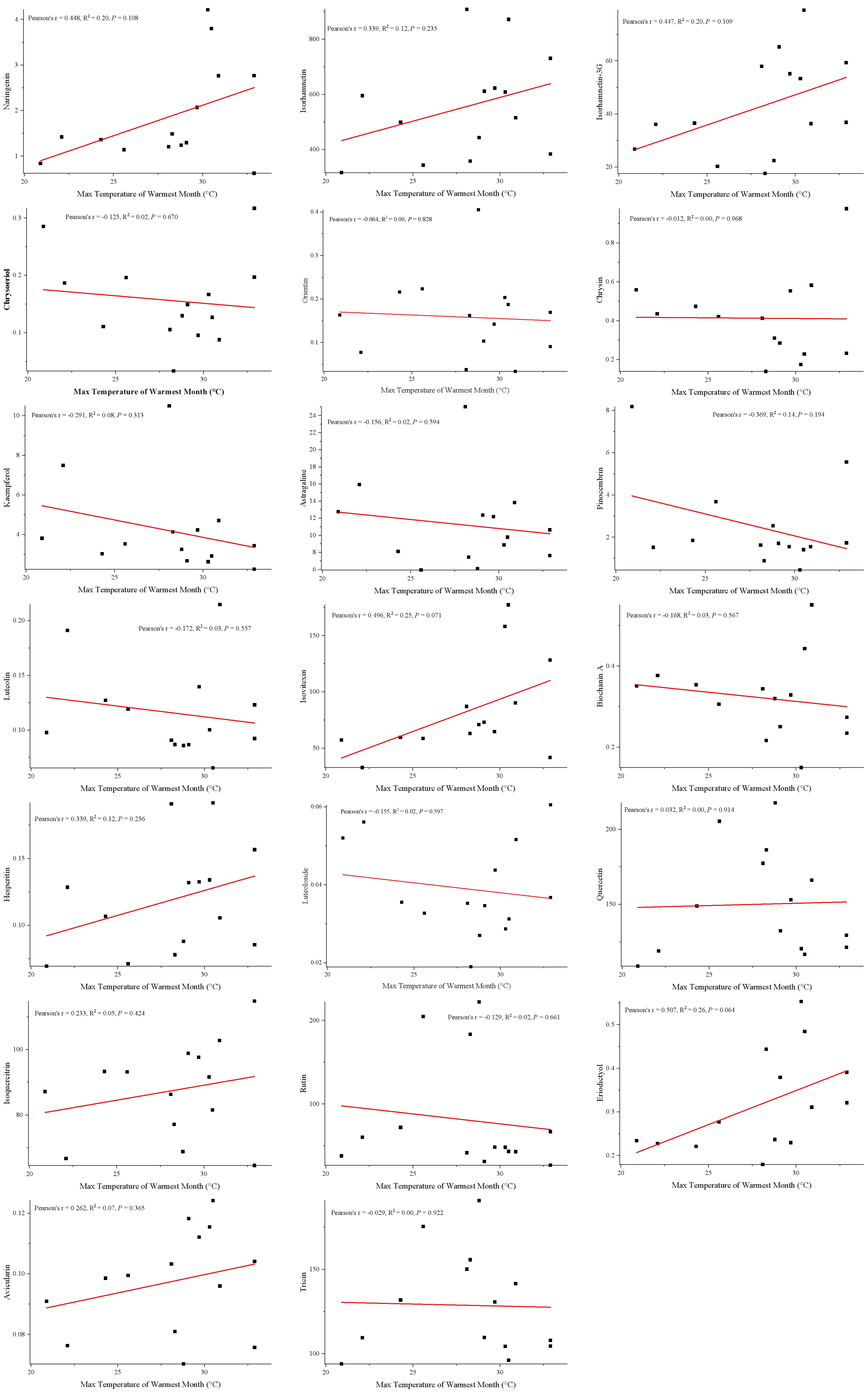

Figure S6 Relationship between max temperature of warmest month and flavonoid metabolites. The linear regressions were considered significant when $P < 0.05$.

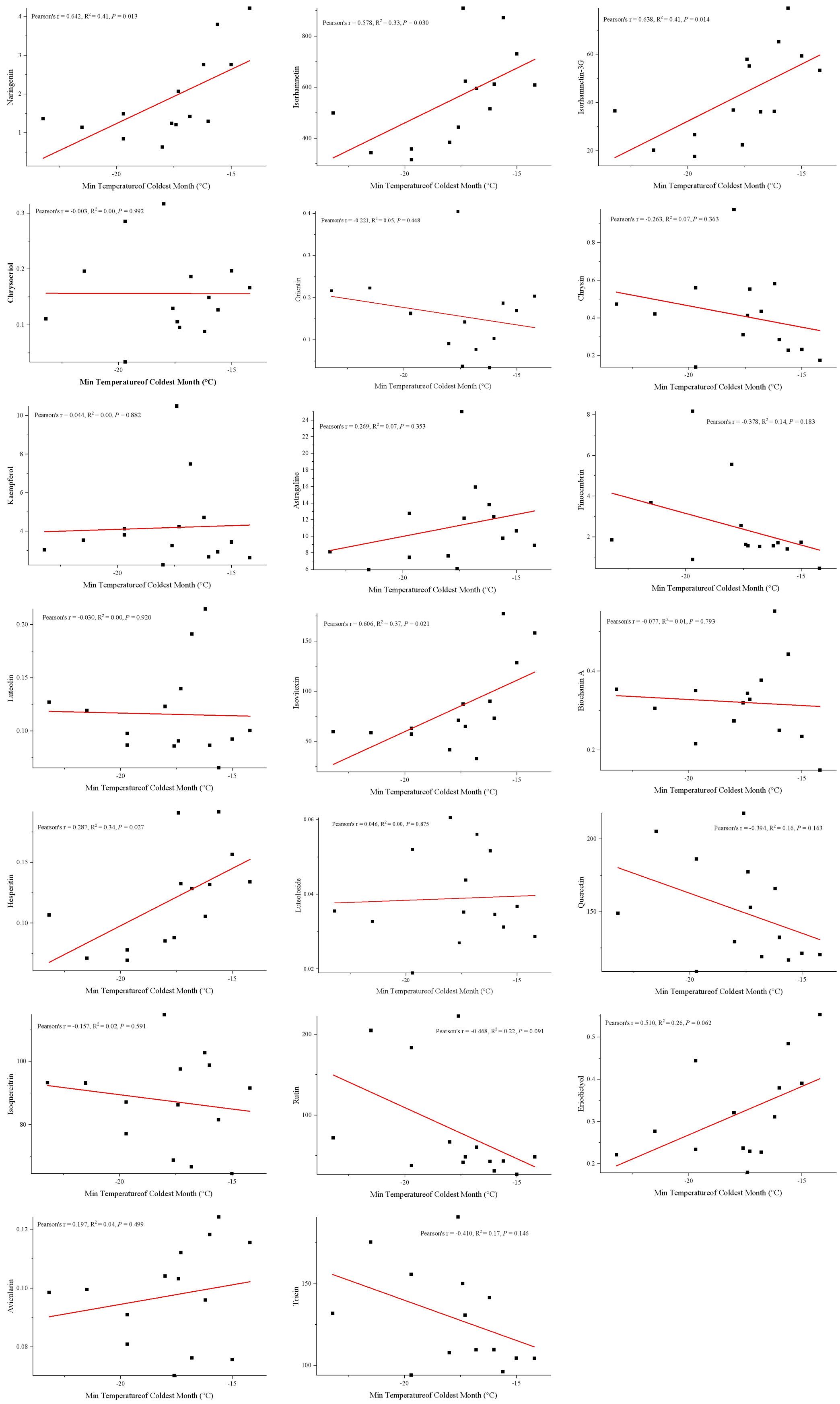

Figure S7 Relationship between temperature of coldest month and flavonoid metabolites. The linear regressions were considered significant when $P < 0.05$.

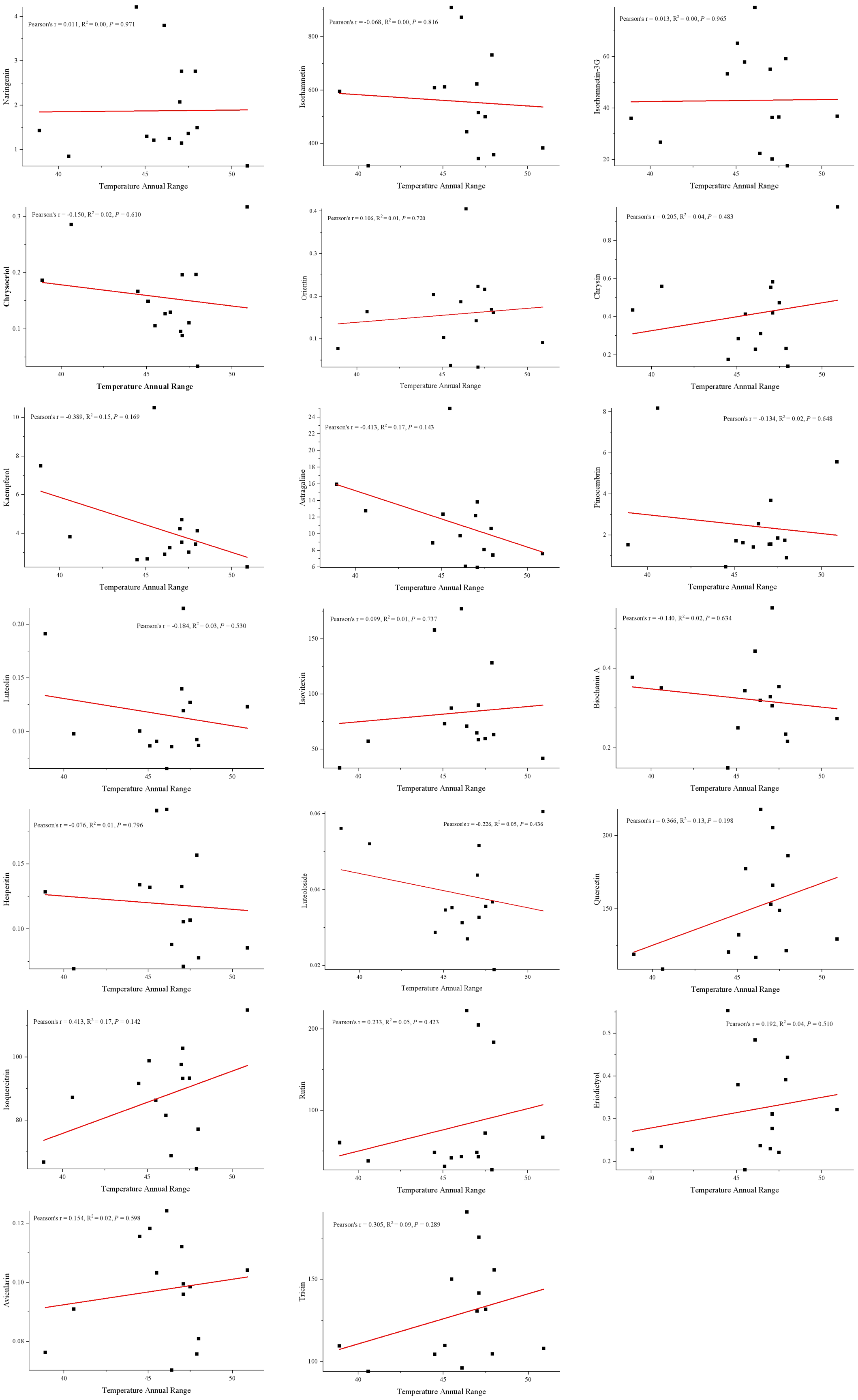

Figure S8 Relationship between temperature annual range and flavonoid metabolites. The linear regressions were considered significant when $P < 0.05$.

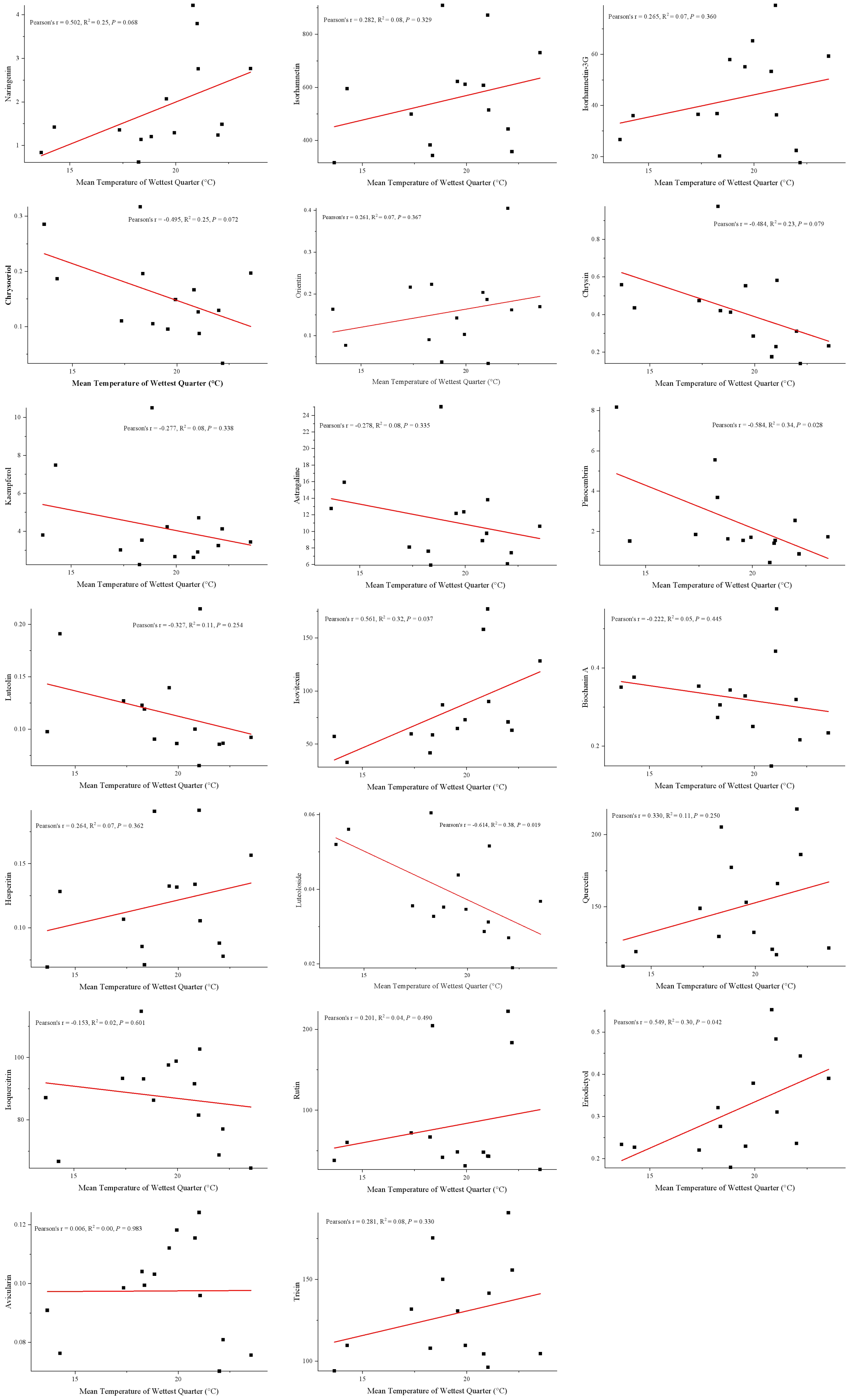

Figure S9 Relationship between mean temperature of wettest quarter and flavonoid metabolites. The linear regressions were considered significant when $P < 0.05$.

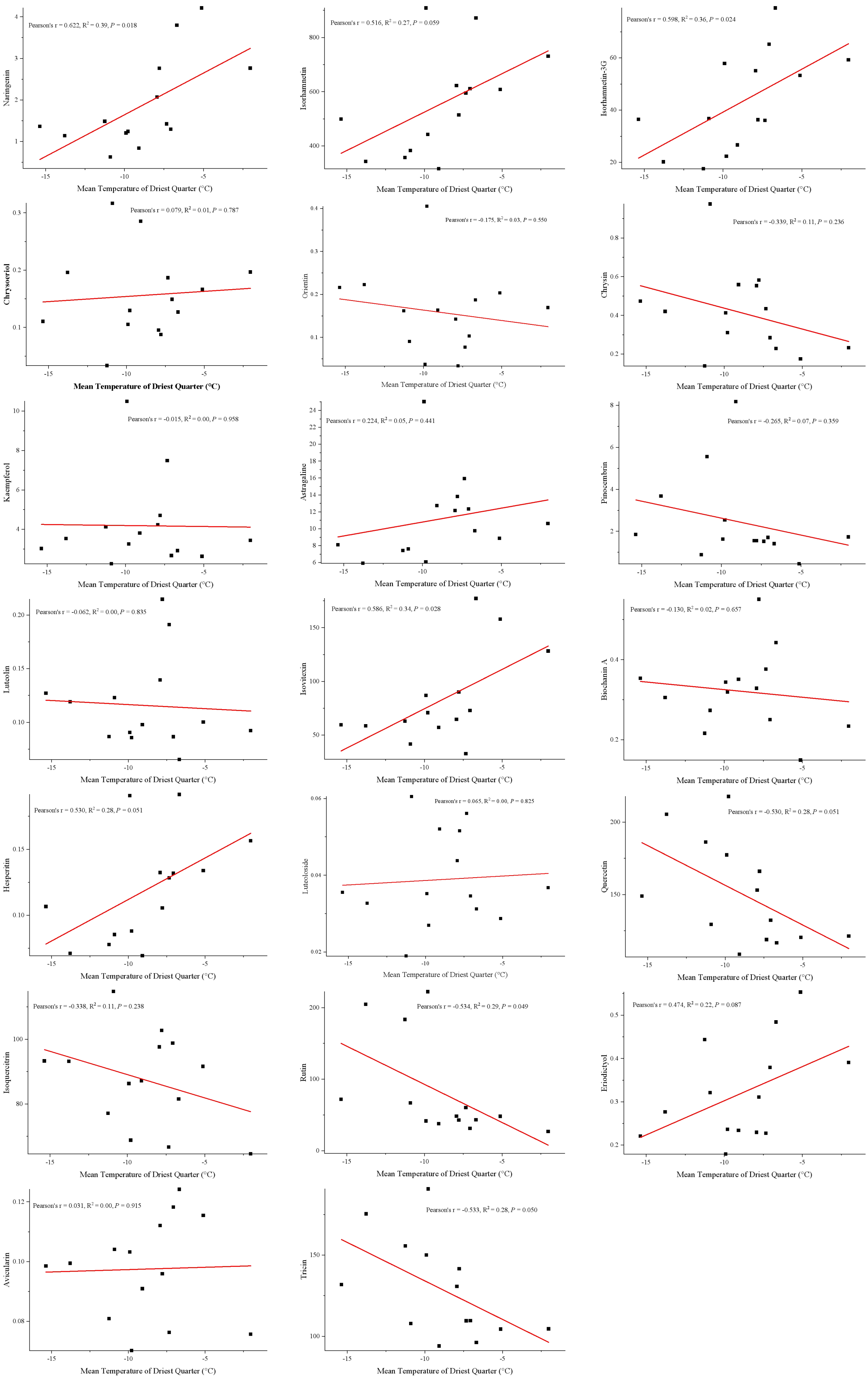

Figure S10 Relationship between mean temperature of driest quarter and flavonoid metabolites. The linear regressions were considered significant when $P < 0.05$.

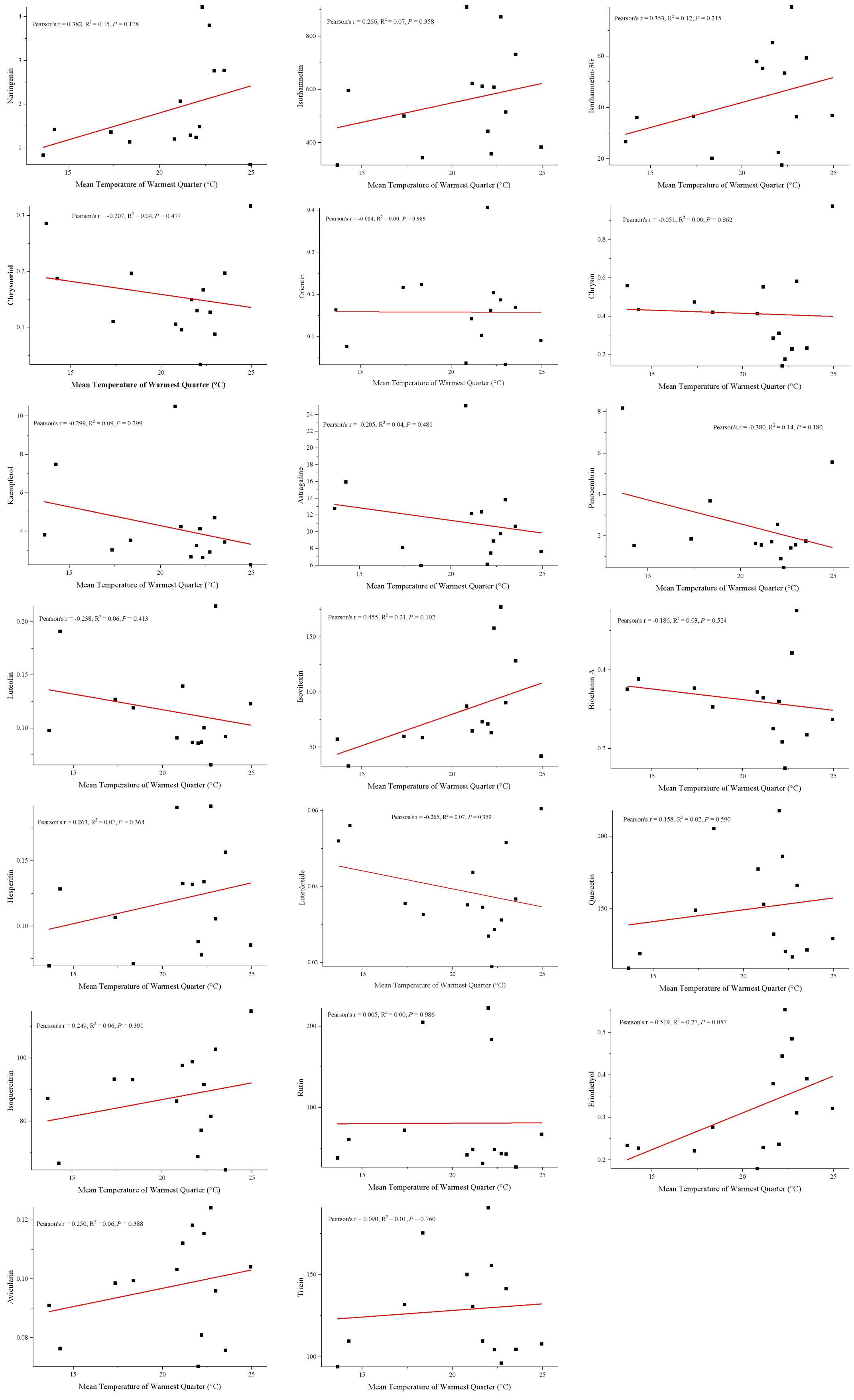

Figure S11 Relationship between mean temperature of warmest quarter and flavonoid metabolites. The linear regressions were considered significant when $P < 0.05$.

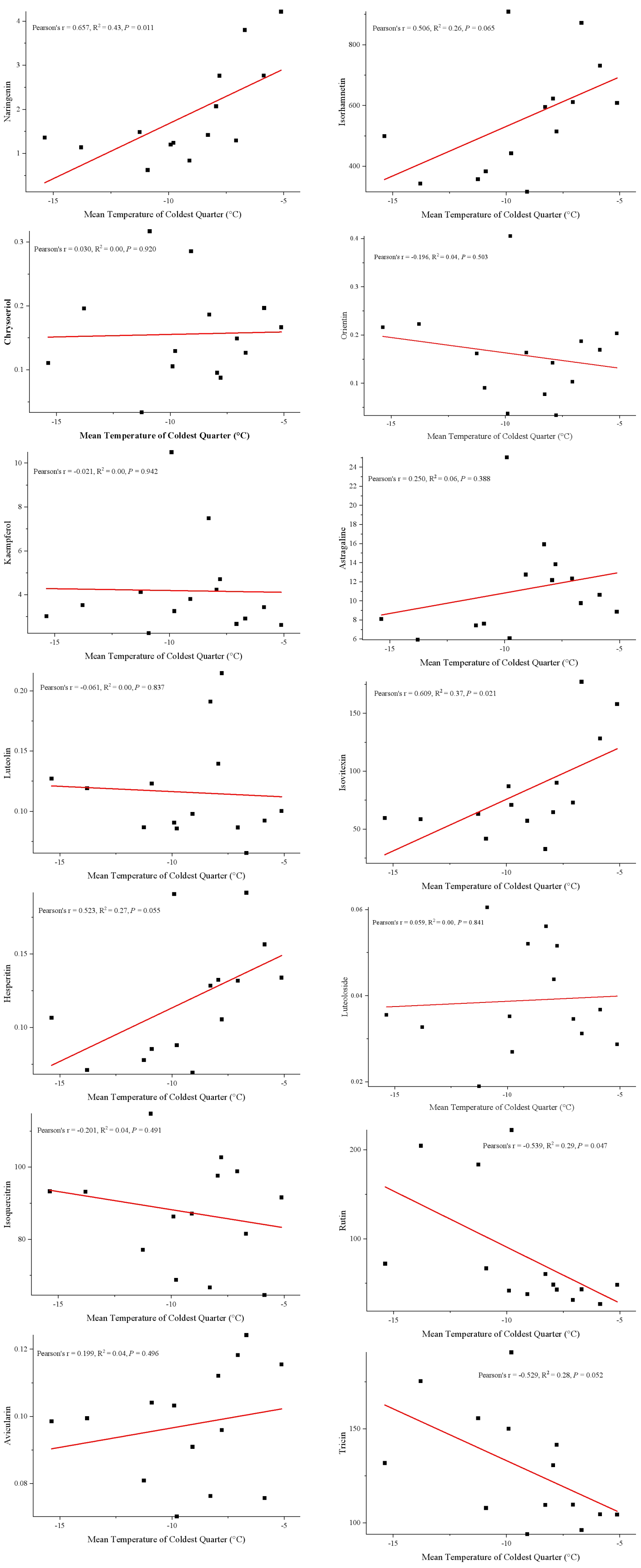

Figure S12 Relationship between mean temperature of coldest quarter and flavonoid metabolites. The linear regressions were considered significant when $P < 0.05$.

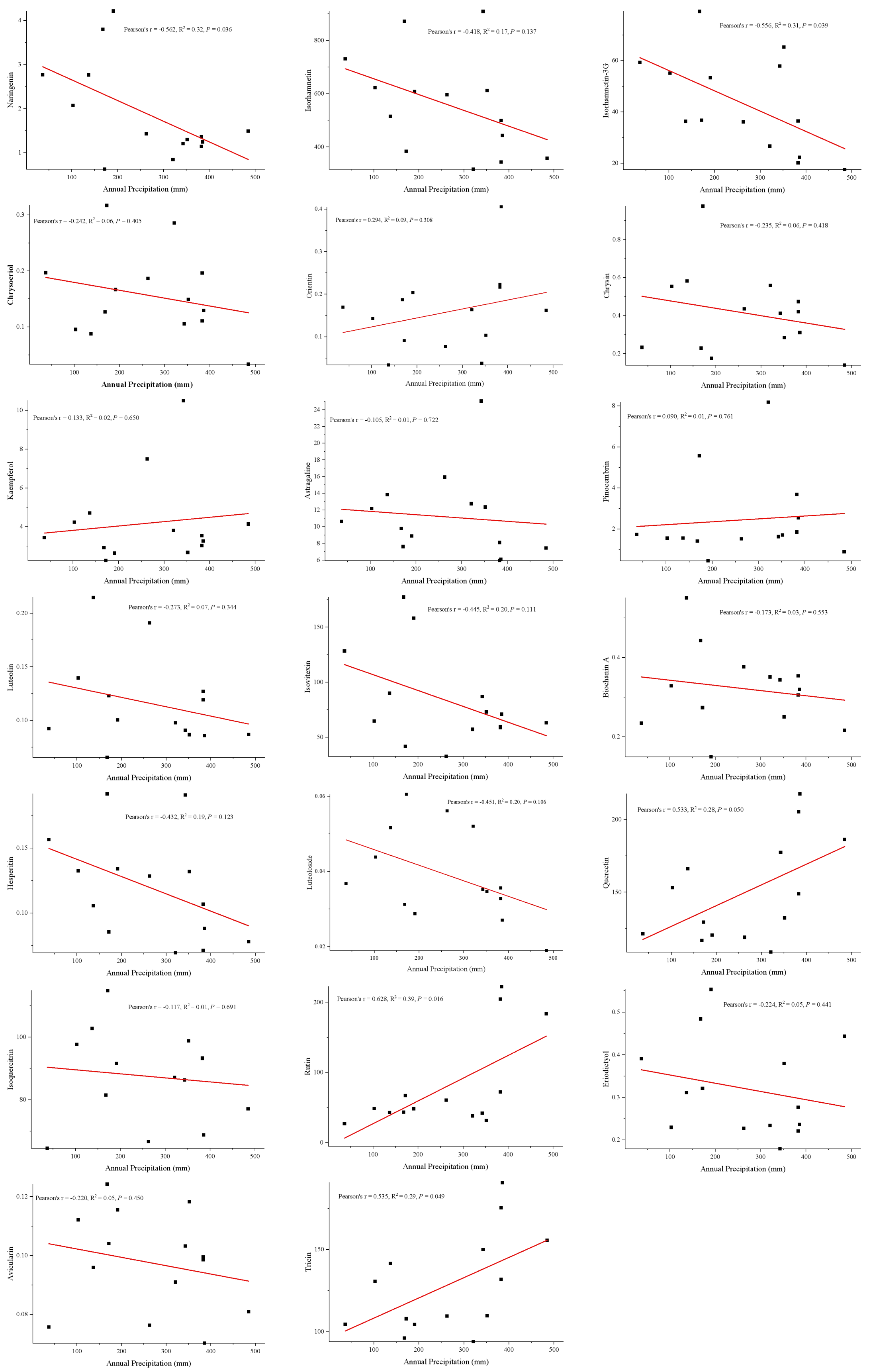

Figure S13 Relationship between annual precipitation and flavonoid metabolites. The linear regressions were considered significant when $P < 0.05$.

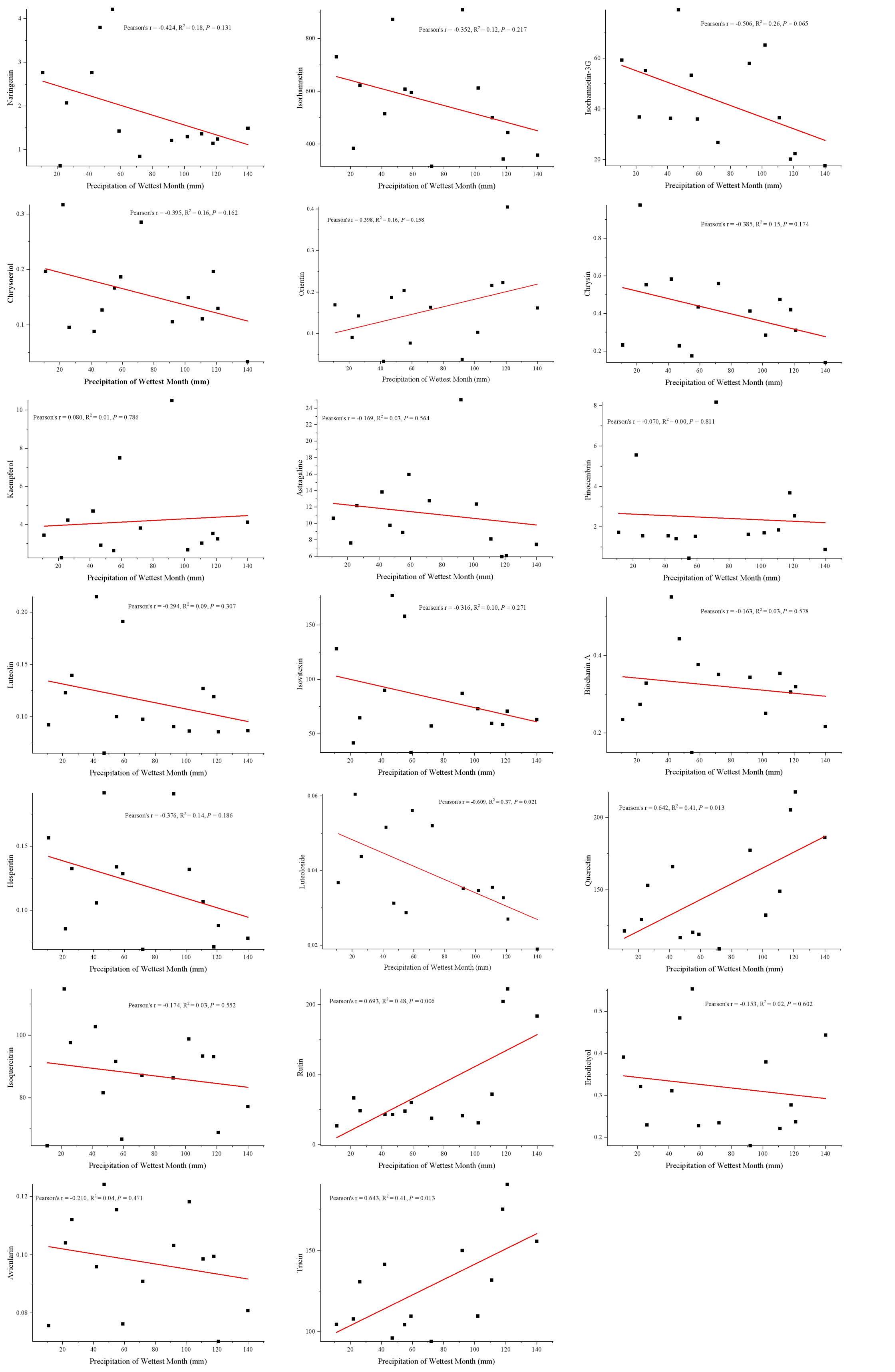

Figure S14 Relationship between precipitation of wettest month and flavonoid metabolites. The linear regressions were considered significant when $P < 0.05$.

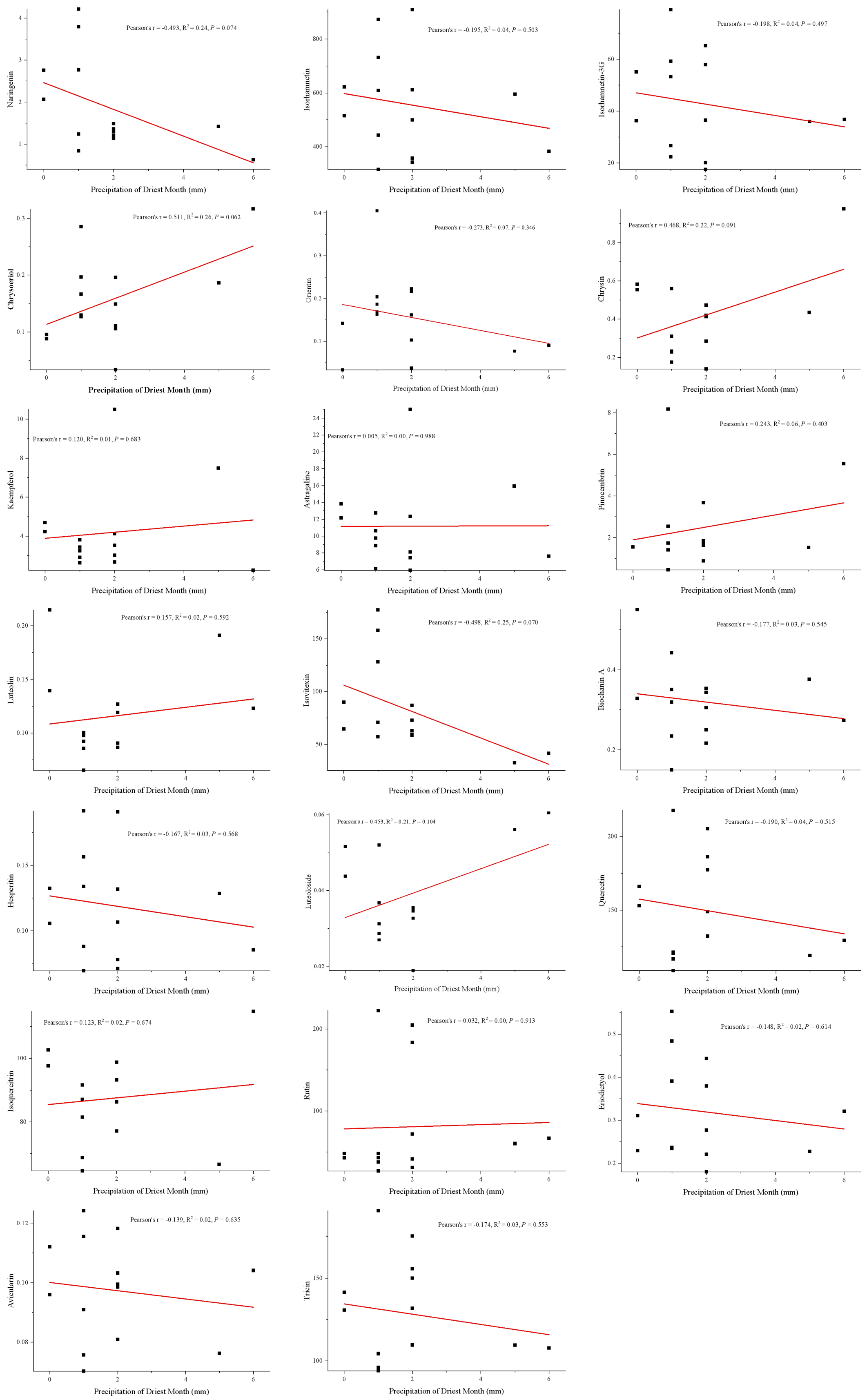

Figure S15 Relationship between precipitation of driest month and flavonoid metabolites. The linear regressions were considered significant when $P < 0.05$.

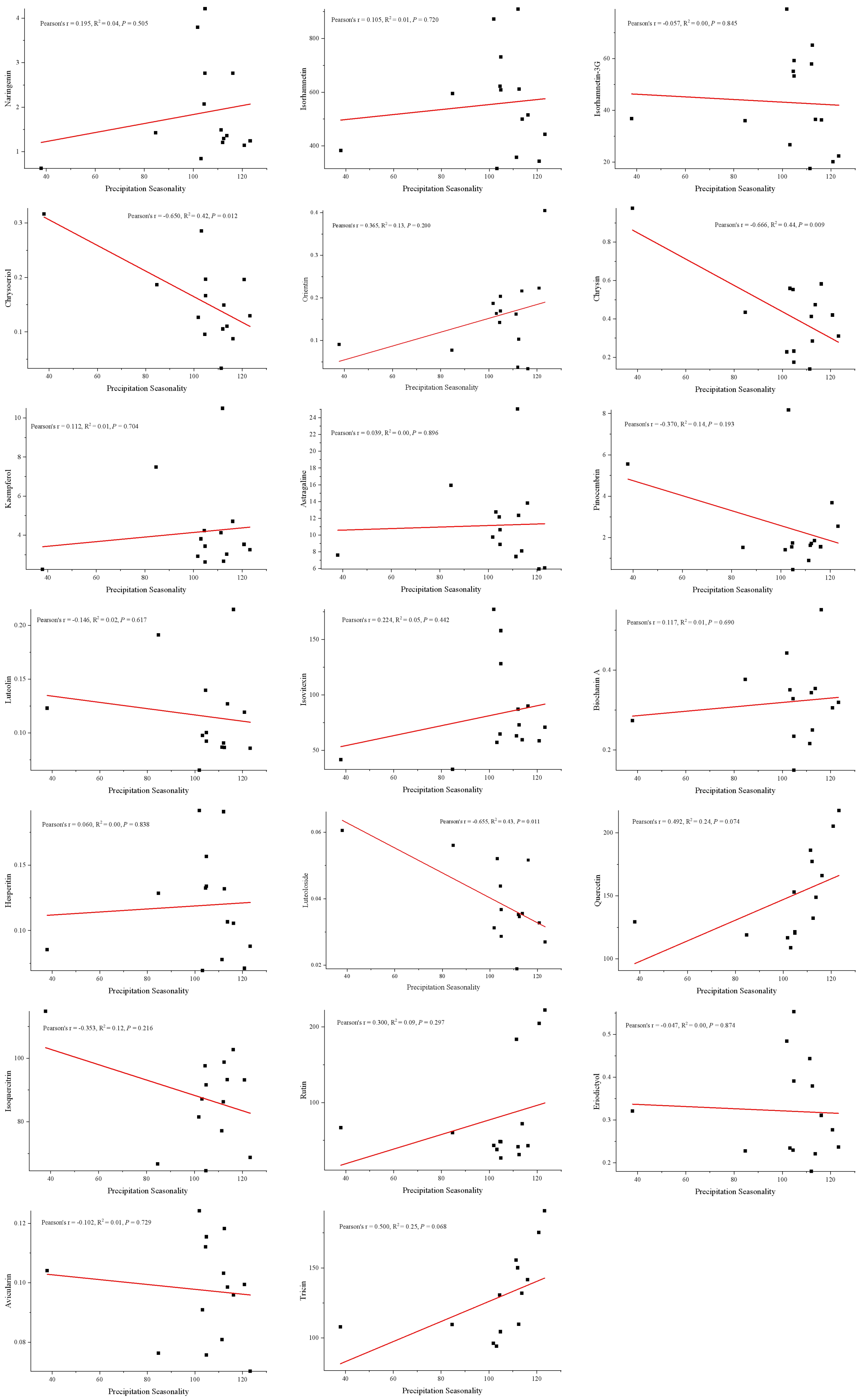

Figure S16 Relationship between precipitation seasonality and flavonoid metabolites. The linear regressions were considered significant when $P < 0.05$.

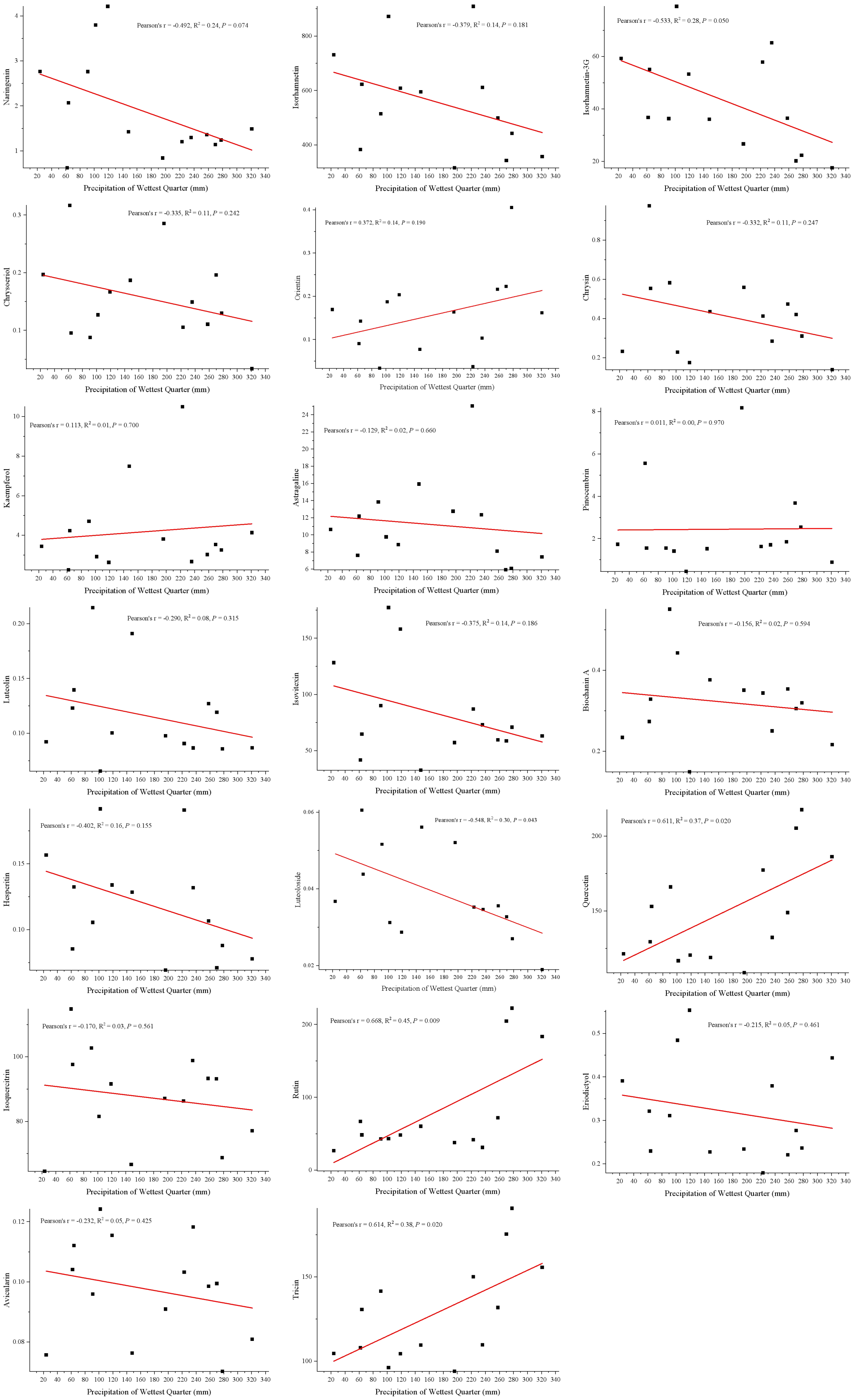

Figure S17 Relationship between precipitation of wettest quarter and flavonoid metabolites. The linear regressions were considered significant when $P < 0.05$.

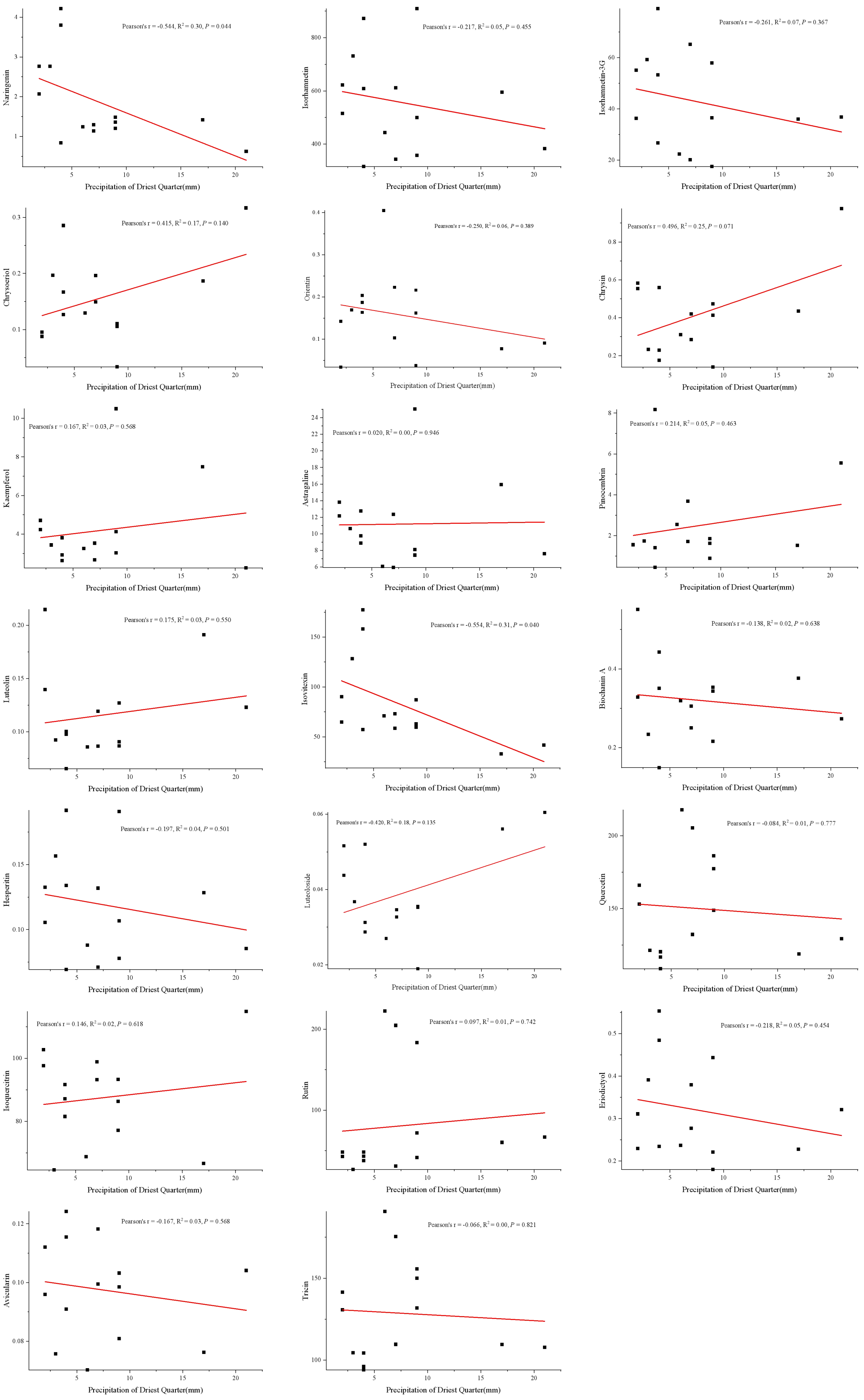

Figure S18 Relationship between precipitation of driest quarter and flavonoid metabolites. The linear regressions were considered significant when $P < 0.05$.

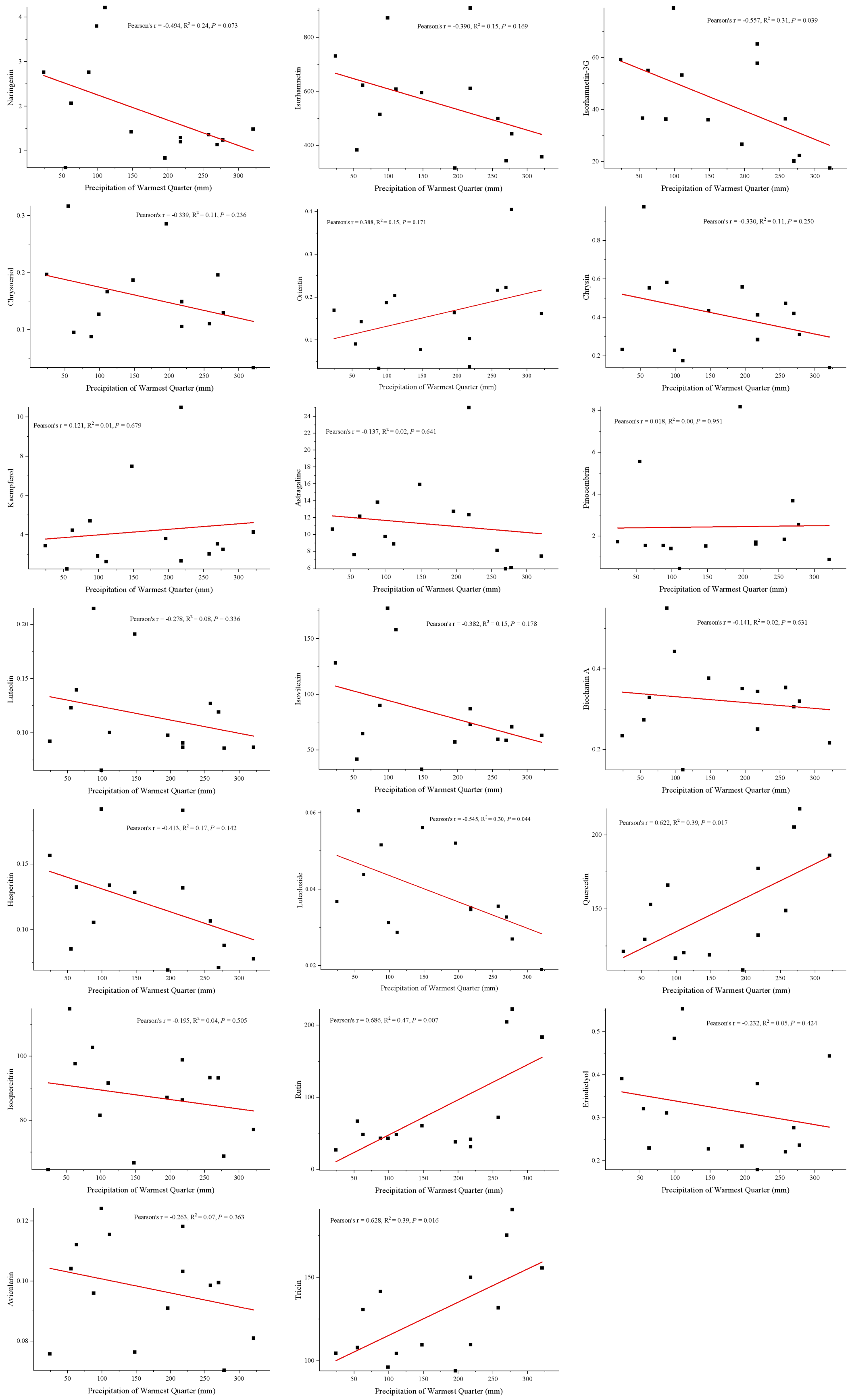

Figure S19 Relationship between precipitation of warmest quarter and flavonoid metabolites. The linear regressions were considered significant when $P < 0.05$.

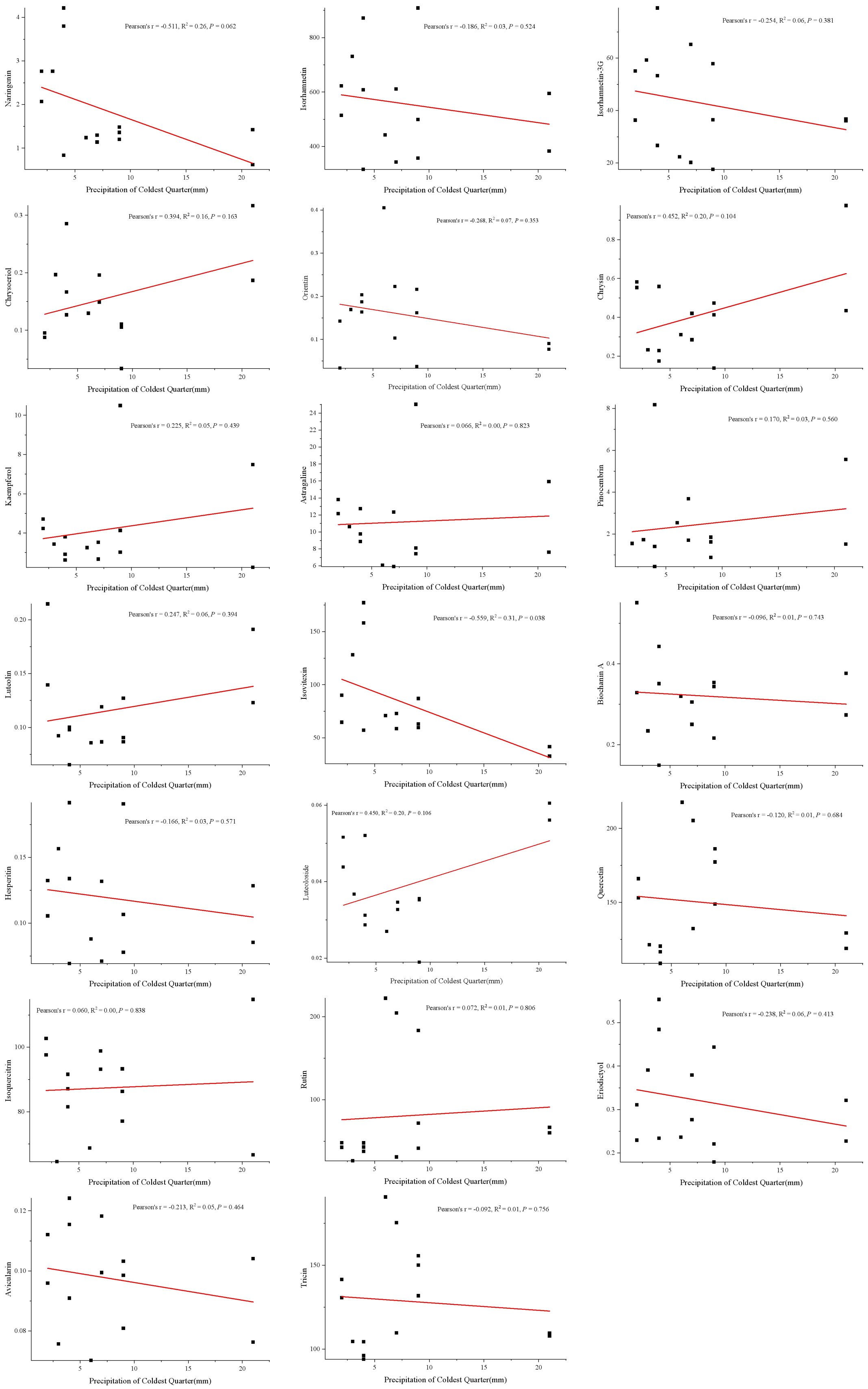

Figure S20 Relationship between precipitation of coldest quarter and flavonoid metabolites. The linear regressions were considered significant when $P < 0.05$.

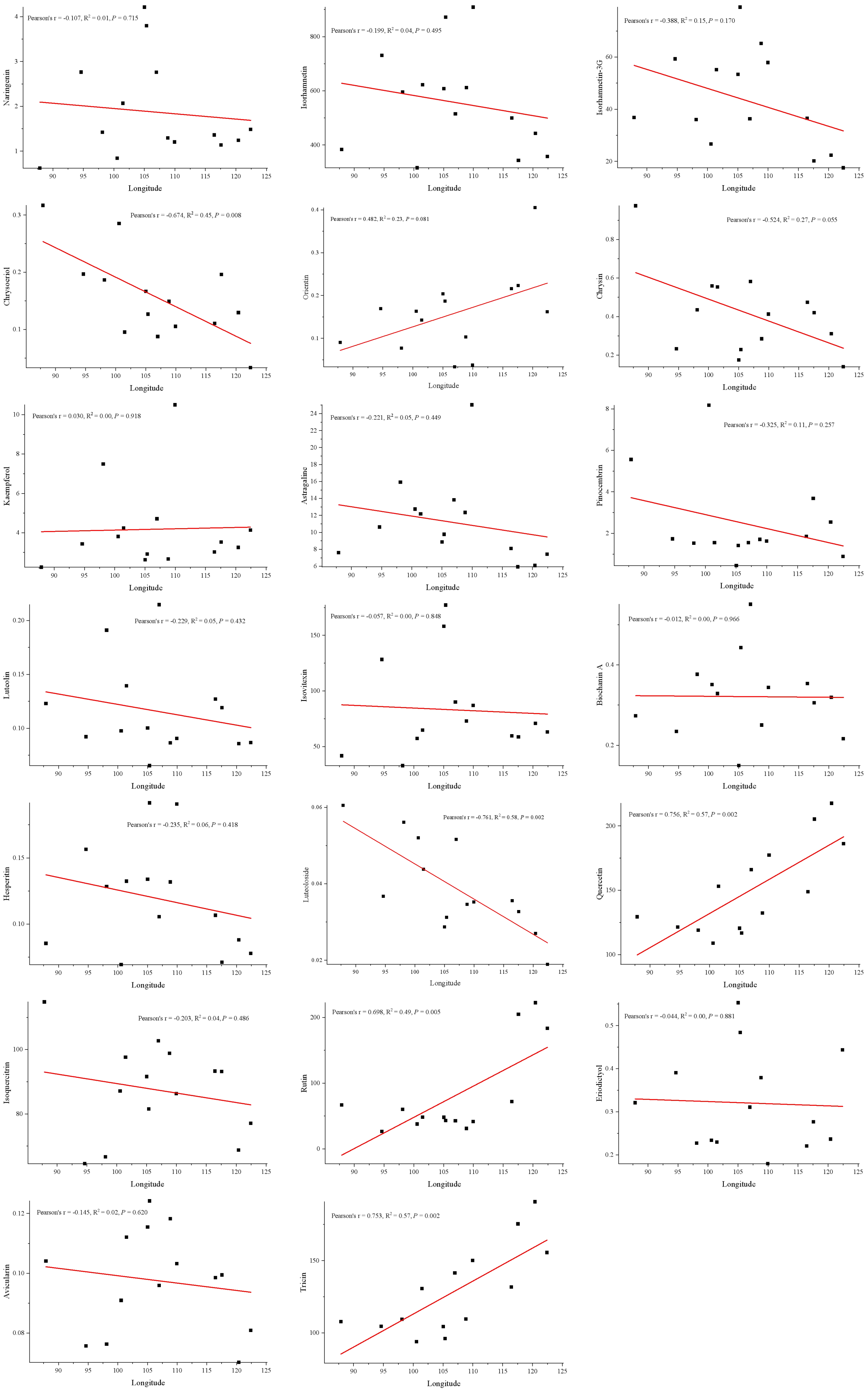

Figure S21 Relationship between longitude and flavonoid metabolites. The linear regressions were considered significant when $P < 0.05$.

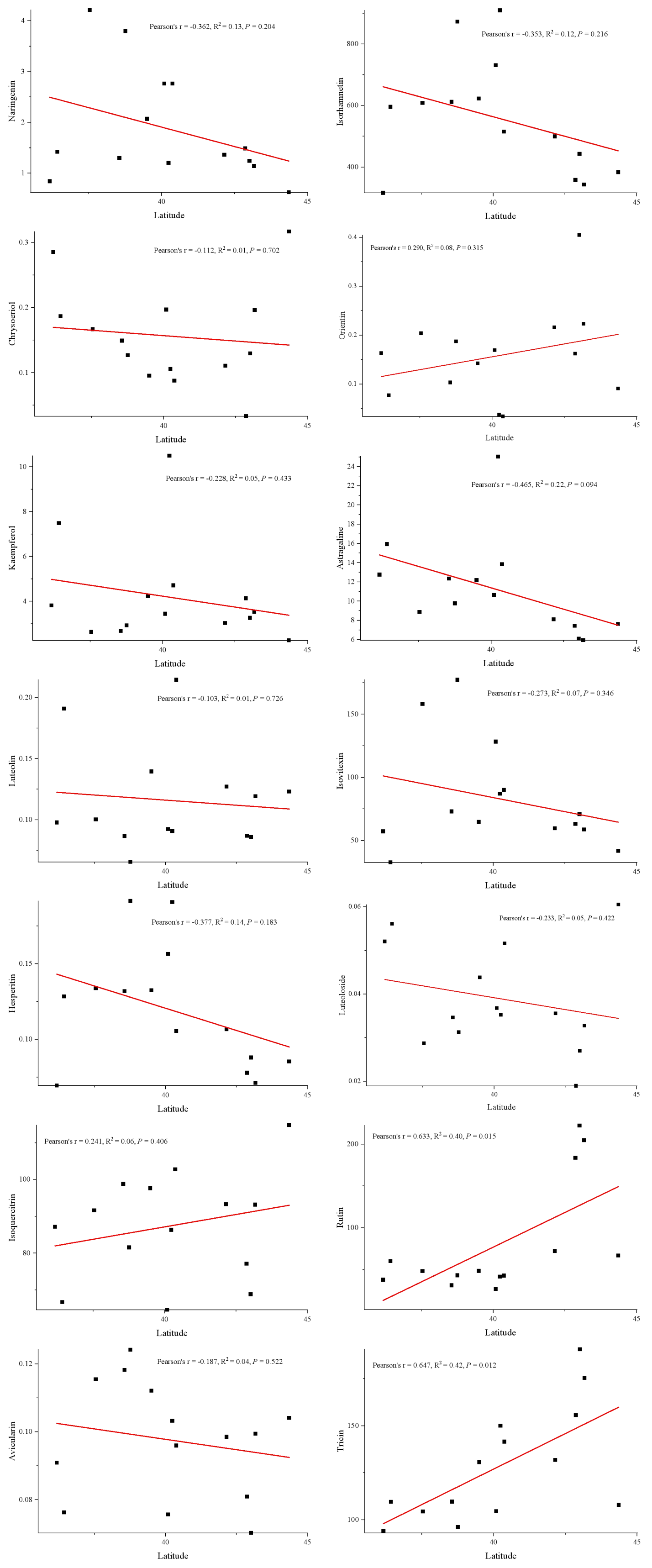

Figure S22 Relationship between latitude and flavonoid metabolites. The linear regressions were considered significant when $P < 0.05$.
